# Transcutaneous Vagus Nerve Stimulation May Enhance Only Specific Aspects of the Core Executive Functions. A Randomized Crossover Trial

**DOI:** 10.3389/fnins.2020.00523

**Published:** 2020-05-25

**Authors:** Uirassu Borges, Laura Knops, Sylvain Laborde, Stefanie Klatt, Markus Raab

**Affiliations:** ^1^Institute of Psychology, German Sport University, Cologne, Germany; ^2^Institute of Clinical Neuroscience and Medical Psychology, Heinrich Heine University, Duesseldorf, Germany; ^3^UFR STAPS, Université de Caen Normandie, Caen, France; ^4^Institute of Exercise Training and Sport Informatics, German Sport University, Cologne, Germany; ^5^Institute of Sports Science, University of Rostock, Rostock, Germany; ^6^School of Applied Sciences, London South Bank University, London, United Kingdom

**Keywords:** tVNS, vagus nerve stimulation, HRV, heart rate variability, cardiac vagal activity, executive functions, neurovisceral integration model

## Abstract

**Background:**

Individuals are able to perform goal-directed behaviors thanks to executive functions. According to the neurovisceral integration model, executive functions are upregulated by brain areas such as the prefrontal and cingulate cortices, which are also crucially involved in controlling cardiac vagal activity. An array of neuroimaging studies already showed that these same brain areas are activated by transcutaneous vagus nerve stimulation (tVNS). Despite evidence toward effects of tVNS on specific executive functions such as inhibitory control, there have been no studies investigating what type of inhibition is improved by tVNS by systematically addressing them within the same experiment. Furthermore, the effect of tVNS on another core executive function, cognitive flexibility, has not yet been investigated.

**Objective:**

We investigated the effects of tVNS on core executive functions such as inhibitory control and cognitive flexibility.

**Methods:**

Thirty-two participants (nine women, *M*_*age*_ = 23.17) took part in this study. Vagally mediated heart rate variability parameters (root mean square of successive differences, RMSSD, and high frequency, HF) were measured while participants performed four different cognitive tasks that mainly rely on different aspects of both the aforementioned executive functions.

**Results:**

Despite clear conflict effects in the four tasks, only performance on the task used to measure set-shifting paradigm was improved by tVNS, with switch costs being lower during tVNS than during sham stimulation. Furthermore, HF increased during each of the cognitive flexibility tasks, although HF during tVNS did not differ from HF during sham stimulation.

**Conclusion:**

The results indicate for the first time (a) that tVNS can increase cognitive flexibility in a set-shifting paradigm, and (b) that tVNS may exert a stronger effect on cognitive flexibility than inhibition. The present study provides only partial evidence for the neurovisceral integration model. Future studies should address further paradigms that demand cognitive flexibility, thus investigating this new hypothesis on the specificity of the tVNS effects on cognitive flexibility.

## Introduction

Transcutaneous vagus nerve stimulation (tVNS) is a technology used to electrically and non-invasively modulate vagal activity through the auricular branch of the vagus nerve. There has been an increasing amount of studies using tVNS to enhance cognitive processes that rely on prefrontal activity. An array of these studies addressed specific aspects of inhibitory control separately (e.g., Keute et al., 2018a; Ventura-Bort et al., 2018), whereas others investigated more complex cognitive functioning such as creativity (Colzato et al., 2018b) and implicit spiritual self-representation (Finisguerra et al., 2019). Attempts motivated by theory-driven hypotheses to systematically investigate the effects of tVNS on different aspects of basic cognitive functions are still scarce. Based on the predictions outlined in the neurovisceral integration model (Thayer et al., 2009), the current study aimed at investigating the effects of tVNS on the core executive functions inhibitory control and cognitive flexibility (Diamond, 2013). Furthermore, and also in line with the neurovisceral integration model, we measured cardiac vagal activity during tVNS and cognitive performance, a parameter suggested to reflect the effectiveness of executive functioning.

Executive functions refer to top–down mental processes that serve goal-directed behavior (Diamond, 2013). Inhibitory control and cognitive flexibility are considered core executive functions, meaning that they are necessary components for building higher-order executive functions (Miyake and Friedman, 2012; Diamond, 2013). Inhibitory control involves the ability to override dominant or prepotent responses by controlling one’s attention and behavior, and can be distinguished between selective attention and response inhibition (Diamond, 2013). Selective attention is expressed by the inhibitory cognitive control of attention, which occurs by suppressing prepotent mental representations on the level of perception. Response inhibition is a behavioral inhibition that keeps a person from acting impulsively. Cognitive flexibility consists in quickly and flexibly switching between tasks or mental sets (Diamond, 2013). It can be broken down into task switching and set shifting. Task switching differs from set shifting in the type of conflict: task switching is related to switching between tasks with different instructions involving different stimuli. Set shifting, in turn, consists of shifting attention between different features of the same stimuli to follow a given instruction (Dajani and Uddin, 2015).

Executive functioning is linked to prefrontal activity (Arnsten and Li, 2004). According to the neurovisceral integration model (Thayer et al., 2009; Smith et al., 2017), cardiac vagal activity–the activity of the vagus nerve regulating cardiac functioning–reflects the output of the central autonomic network, which links the prefrontal cortex to the heart (Thayer et al., 2009). The optimal activation of the neural pathways within this network is crucial for performing a given task that requires cognitive effort and for showing flexible responses to a changing environment (Thayer et al., 2009). Because cardiac vagal activity and executive functioning share common underlying neurovisceral self-regulation mechanisms, higher cardiac vagal activity is associated with improved executive functioning. Cardiac vagal activity can be indexed via heart rate variability (HRV), the difference in the time interval between adjacent heartbeats (Malik et al., 1996), and specifically by the root mean square of the successive differences (RMSSD) and by high frequency (HF).

There is a large body of empirical evidence linking higher levels of cardiac vagal activity to higher executive performance (Inhibitory control: Alderman and Olson, 2014; cognitive flexibility: Johnsen et al., 2003; Colzato et al., 2018a). Based on the evidence of the relationship between executive functioning and cardiac vagal activity as indexed by HRV (RMSSD and HF), in the present study we will consider the executive functions described here to investigate if tVNS can improve different types of inhibitory control and cognitive flexibility as well as cardiac vagal activity.

The expected link between tVNS and executive functions can be understood by considering the neuroanatomical pathways of the vagus nerve. The electrical signal, starting in the auricular branch of the vagus nerve (ABVN), reaches the nucleus tractus solitarius, which is a crucial structure that projects to a variety of brain areas, including cortical regions such as the anterior cingulate cortex and the prefrontal cortex (Aihara et al., 2007). As shown by several functional magnetic resonance imaging (fMRI) studies (Kraus et al., 2013; Frangos et al., 2015; Yakunina and Kim, 2017; Badran et al., 2018), tVNS evoked, in contrast to sham stimulation, higher activity in the nucleus tractus solitarius (Frangos et al., 2015; Yakunina and Kim, 2017), in the left prefrontal cortex and in cingulate areas (Badran et al., 2018). Importantly, these brain areas affected by tVNS correspond to the areas described by the neurovisceral integration model as regulating both executive and cardiac regulation, such as the prefrontal cortex and cingulate areas (Thayer et al., 2009, 2012).

So far, there are studies showing that tVNS affects the types of inhibitory control (Table 1). These studies used varying cognitive paradigms, which comprise different dependent variables, and addressed the inhibitory control types only separately and in different study designs (see Table 1 for an overview of design-related characteristics of studies investigating inhibitory control using tVNS). Thus, an integrating, evidence-based discussion on the interplay between tVNS and these types of inhibitory control has not been possible.

**TABLE 1:** Summary of the studies with tVNS addressing different types of inhibitory control.

**Study**	**Dependent variable**	**Cognitive paradigm**	**Study design**	**Sample size**	**Results**
Beste et al., 2016	Response inhibition and working memory	Backward inhibition and mental workload inhibition paradigm	Between-subjects	51	Higher response inhibition processes only when working memory processes are needed
Fischer et al., 2018	Selective attention, N2 and P3 amplitudes	Simon	Within-subject	21	Adaptation to conflict was enhanced, N2 amplitude higher
Keute et al., 2019a	Automatic motor response inhibition, readiness potentials	Subliminal motor priming	Within-subject	16	Increased NCE; effects on readiness potentials only in compatible trials
Steenbergen et al., 2015	Response selection as a consequence of response inhibition	Stop-change	Between-subjects	30	Faster responses when two actions were executed in succession
Ventura-Bort et al., 2018	Selective attention, sAA, P3a and P3b amplitudes	Oddball	Within-subject	20	Increased sAA after tVNS; easy trials produced larger P3b amplitudes

*NCE, negativity comparability effect; sAA, salivary alpha-amylase; tVNS, transcutaneous vagus nerve stimulation.*

As stated above, executive functions and cardiac vagal activity share overlapping neurological structures, with both being upregulated by cortical areas, including the prefrontal cortex (Thayer et al., 2009). Given that the tVNS signal is sent afferently to the prefrontal cortex via ABVN, cardiac vagal activity has also been thought to be affected by tVNS (Murray et al., 2016). Using RMSSD to measure the effect of tVNS on cardiac vagal activity, different studies did not find any differences between active and sham stimulation (Burger et al., 2016, 2019; De Couck et al., 2017). One study showed in three experiments that tVNS consistently increased RMSSD; however, this increase was similarly observed during both active and sham stimulation, with this possibly indicating that tVNS sends non-specific signals at the brainstem level that similarly influence cardiac vagal activity in both active and sham stimulation (Borges et al., 2019). Nonetheless, this study did not take any cognitive paradigm into account, which might have contributed to understanding if this possible signal non-specificity-identified as an increase in cardiac vagal activity during both active and sham stimulation-can also be observed in cognitive functions. This possibility would challenge the use of earlobe sham stimulation, which has widely been used in current research with tVNS. Therefore, further studies on the effect of active as well as sham tVNS on cardiac vagal activity are still needed.

To summarize, there is evidence toward the modulation of inhibitory control by tVNS; however, these findings refer to different cognitive phenomena that have been found in different samples and in the context of different study designs. So far, there is no study that has systematically investigated the effects of tVNS on different aspects of core executive functions, and importantly, there is a lack of studies whose hypotheses were explicitly motivated by a theory. To address different aspects of executive functioning in an integrative way, it is crucial to use the same study design and setup. This way it is possible to control for possible experimental variations such as length of resting and of stimulation periods, daytime, and other factors that might influence measurement of cardiac vagal activity. Confounders related to study design, e.g., instructions, laboratory setup, and differences in sample size, can also be considered. Thus, going beyond existing literature, the present study aims at investigating the effects of tVNS on inhibitory control, cognitive flexibility, and cardiac vagal activity. To achieve this, it uses an integrative theoretical background, namely the neurovisceral integration model (Thayer et al., 2009), and applies the same study design across these target executive functions. Based on the evidence on neurophysiological pathways related to tVNS, addressing cognitive processes that mainly rely on different executive functions might help to further understand how tVNS affects basic cognitive processes involved in goal-directed behavior.

Against this background, it was hypothesized that the performance on the four cognitive tasks is higher during active tVNS, compared to sham stimulation (H1a for selective attention, H1b for response inhibition, H1c for task switching, and H1d for set shifting; this assignment of the subtypes of executive functions to the letters is also valid for the next hypotheses). Furthermore, we expected that cardiac vagal activity increases relatively to the resting phase only during active stimulation and not during sham stimulation, with cardiac vagal activity during the tasks being higher in the active tVNS condition (H2a–d). Moreover, we hypothesized that cardiac vagal activity during tVNS and before each cognitive task is positively associated with task performance only in the active tVNS condition (H3a–d). Finally, we expected cardiac vagal activity during the tasks to have a more strongly positive relationship to task performance in the active condition than in the sham condition (H4a–d).

## Materials and Methods

### Participants

**FIGURE 1:**
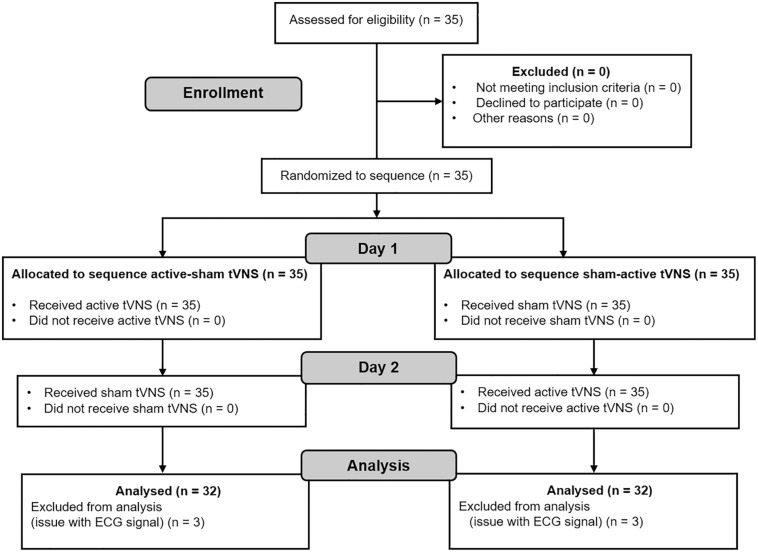
Consort (2019) diagram.

As it is not possible to run power analyses for multi-factorial repeated-measures designs with G^∗^Power 3.1 (Faul et al., 2007), we followed the same procedure found in previous studies with similar study design (e.g., Liepelt et al., 2019). Accordingly, we matched the average number of participants in the studies that investigated executive functions with tVNS using a within-subject design (summarized in Table 1). Since we also measured cardiac vagal activity, we additionally considered the average sample size in Borges et al. (2019), because this study systematically investigated the effect of tVNS on cardiac vagal activity in different experiments. Twenty-nine participants were calculated to be necessary to find an effect. Anticipating possible exclusions due to drop-outs and after data cleaning, we recruited 35 participants. Thirty-two participants (nine female) were included in the analysis due to technical problems with the electrocardiogram (ECG) signal of three participants. Mean age was 23.17 years old (*SD* = 4.08), whereby female participants had *M*_*age*_ = 21.11, *SD* = 1.27, and male participants had *M*_*age*_ = 24.87, *SD* = 5.87. Consort flowchart (Dwan et al., 2019) is presented in Figure 1.

The sample consisted of healthy students at the local university. Participants were eligible if they were not pregnant at the time of the experiment and free of cardiovascular or neurological diseases, or major mental disorders, for example severe depression or anxiety disorder. They were asked not to smoke, exercise, or consume food, alcohol, or caffeine for at least 2 h before participation. These potentially confounding variables as well as tVNS safety-related questions were assessed by means of an adapted version of the demographics questionnaire for experiments using HRV developed by Laborde et al. (2017). All participants gave written informed consent prior to the experiment, which was approved by the local ethical committee (ethics approval number 120/2018).

### Transcutaneous Vagus Nerve Stimulation

We employed the NEMOS tVNS device developed by Cerbomed (Erlangen, Germany). Two titan electrodes found in a structure similar to an earphone are placed in the cymba conchae of the left ear, an area thought to be exclusively innervated by the ABVN (Peuker and Filler, 2002), in order to electrically stimulate these vagal fibers (Ellrich, 2011). In the sham stimulation, the electrodes are placed on the left earlobe, which is thought to be free of vagal innervation (Peuker and Filler, 2002) and has abundantly been used as a sham stimulation in research with tVNS (van Leusden et al., 2015). The tVNS device delivers a stimulation with a pulse width of 200–300 μs at 25 Hz and an on–off cycle of 30 s. Regarding the adjustment of the stimulation intensity, cardiac vagal activity may be similarly influenced by electrical afferent stimuli that are triggered by different methods to stipulate stimulation intensity (Borges et al., 2019). Therefore, we followed procedures found in previous research with tVNS that allow participants to choose their individual intensity (Fischer et al., 2018; Ventura-Bort et al., 2018). Accordingly, in each session participants received increasing and decreasing series of 10-s stimulation trials, and rated the subjective sensation of the stimulation on a 10-point scale, ranging from nothing (0), light tingling (3), strong tingling (6), to painful (10). The increasing series of trials started from an intensity of 0.01 mA and increased by 0.01 mA on a trial-by-trial basis until participants reported a tingling sensation of 9. Before starting the decreasing series, the same intensity was repeated and then reduced trial by trial in 0.01 mA until a subjective sensation of 6 or below was experienced. This procedure was repeated a second time. The final stimulation intensity used for the experimental procedure was calculated based on the average of the four intensities rated as 8 (two from the increasing and two from the decreasing series). The average chosen stimulation intensity in the active condition was *M* = 2.19 mA (*SD* = 0.93) and *M* = 2.20 mA (*SD* = 1.06) in the sham condition. These stimulation intensities did not differ significantly from each other, *t*(31) = 0.063, *p* = 0.950.

### Cardiac Vagal Activity

To assess cardiac vagal activity, we used the Faros 180° device from Mega Electronics (Kuopio, Finland) with a set sampling rate of 500 Hz. This device enables users to measure the ECG signal as recommended by current guidelines on HRV measurement for psychophysiological experiments (Laborde et al., 2017). We placed two disposable ECG pre-gelled electrodes (Ambu L-00-S/25, Ambu GmbH, Bad Nauheim, Germany) on the chest, the positive electrode on the right infraclavicular fossa and the negative one on the left anterior axillary line below the 12th rib.

RMSSD, as well as HF (0.15–0.40 Hz band) transformed with autoregressive modeling, were chosen as indicators of cardiac vagal activity in the main analyses (Malik et al., 1996). From ECG recordings, we extracted HRV with Kubios software (University of Eastern Finland, Kuopio, Finland), visually inspected the full ECG recording, and manually corrected artifacts (Laborde et al., 2017). Since HF is only influenced by breathing when breathing cycles are between nine cycles per minute (0.15 Hz) and up to 24 cycles per minute (0.40 Hz) (Malik et al., 1996), participants with a respiration rate of less than nine cycles per minute and more than 24 cycles per minute were excluded from analyses with HF. The respiration rates (the number of respiratory cycles per minute) was obtained multiplying the ECG-derived respiration value obtained via the Kubios algorithm by 60 (Tarvainen et al., 2013) and was also separately analyzed. We considered for analysis measurements in blocks of 4 min, which is in accordance with the range suggested by recommendations for experiment planning in psychophysiological research (Laborde et al., 2017). Given that the cognitive tasks differed greatly from one another regarding time length, with the tasks lasting between 5 and 13 min, for the analysis within task blocks we chose a time window of the last 4 minutes respectively for each cognitive task.

### Cognitive Tasks

In order to standardize the tasks and therefore avoid response mistakes, all tasks used the keys “S” and “K” as responses for left and right, respectively. The participants were instructed to press the buttons with their index fingers, and the stimuli were presented in white against a gray background (except for the set-shifting task). We used a 24-in. flat-screen monitor (1,920 × 1,080 pixels at 60 Hz) at a viewing distance of 60 cm to present the tasks and ran all of them with PsychoPy3 Version 3.0.0 (Peirce et al., 2019). The participants performed four tasks which are thought to mainly rely on inhibitory control (selective attention and response inhibition), and cognitive flexibility (task switching and set shifting). These tasks were chosen according to two criteria: First, we followed recommendations from influential reviews on executive functions (Miyake and Friedman, 2012; Diamond, 2013). For the choice of the cognitive task, we considered the task impurity problem: according to Miyake and Friedman (2012), because executive functions necessarily manifest themselves by operating on other cognitive processes, any executive task strongly implicates other cognitive processes that are not directly relevant to the target executive function. Consequently, we chose the tasks that are thought to minimize demands of other executive functions (Diamond, 2013). Second, we performed a literature search to find studies that used the tasks recommended by the aforementioned reviews and also provided evidence on the relationship with (a) tVNS, (b) cardiac vagal activity, and (c) prefrontal activity (imaging studies). The tasks chosen are the following:

#### Flanker Task

**FIGURE 2:**
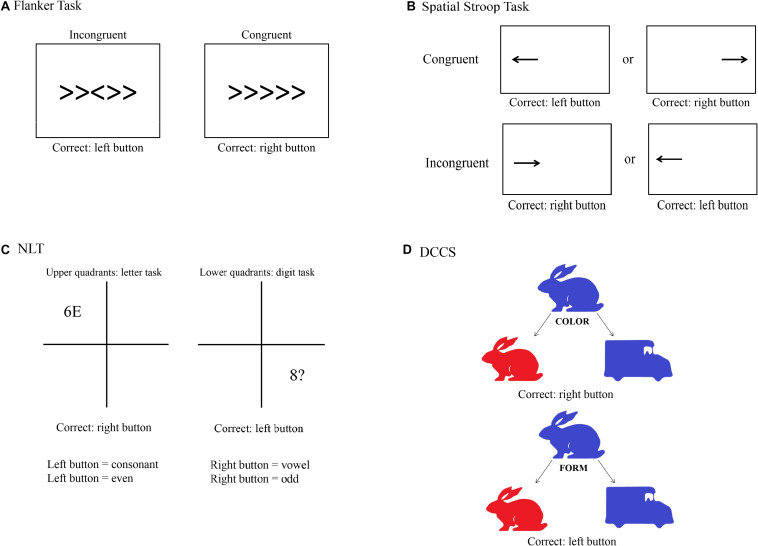
Visual depiction of the four cognitive tasks used in the study. **(A)** Flanker task; **(B)** Spatial Stroop task; **(C)** Number–Letter task (NLT); **(D)** Dimensional Change Card Sorting task (DCCS).

Following recommendations from Diamond (2013), to measure selective attention we used a modified version of the Flanker task (Eriksen and Eriksen, 1974). We used the Flanker task as reported by Alderman and Olson (2014). With this version, it could be shown that individuals with higher fitness levels expressed higher HF values during the task, and that these individuals had lower RT than the less fit group. A trial consists of five arrows in which the third one is the target arrow. Participants were asked to press the left key on the computer keyboard when the target arrow pointed to the left and the right key when the target arrow pointed to the right. Participants were instructed to respond as quickly and accurately as possible for each trial. After a practice block of 30 trials, two experimental blocks of 120 trials each were presented, each separated by 30 s. Each block consisted of congruent and incongruent stimuli presented in random order. The congruent trials consisted of the target arrow being flanked by arrows facing the same direction, while incongruent trials involved the target arrow being flanked by arrows facing the opposite direction. Each stimulus was presented for 100 ms (to increase task difficulty) with a response window of 1,500 ms. A random inter-stimulus time interval of 1,100, 1,300, or 1,500 ms was also used between each 50 ms visual fixation (+) and the stimulus in order to increase task difficulty (Figure 2A).

#### Spatial Stroop Task

The task for measuring response inhibition was the Spatial Stroop task, as this task is thought to minimize memory demands compared to other classical tasks such as the Simon task (Diamond, 2013). This response inhibition task was designed according to Marotta et al. (2018), from which we only took the arrow part of the task, and consisted of a practice and two experimental blocks. During the practice block, 15 trials were presented, and feedback was provided. The practice block was followed by two experimental blocks of 64 experimental trials each. Participants were instructed to fixate a fixation cross presented in the center of the screen. A directional arrow appears randomly on the left or on the right side of the fixation point, and this arrow points randomly to the right or the left side. Participants are required to indicate the direction of the arrow by pressing the left key if the arrow points to the left and the right key if the arrow points to the right, while ignoring its location. They were instructed to respond as quickly and accurately as possible for each trial. The arrow was presented either left or right of the fixation cross for 2,000 ms. Feedback for incorrect key presses was provided to participants in the form of a 220-Hz tone presented for 1,500 ms. This design produced trials that were congruent (e.g., a right-indicating target presented on the right) or incongruent (e.g., a left-indicating target presented on the right, see Figure 2B).

#### Number–Letter Task

We used the Number–Letter task (NLT) as described in Colzato et al. (2018a), which found that participants with higher resting-state cardiac vagal activity showed greater flexibility than individuals with lower resting-state cardiac vagal activity. Throughout the task, a 10-cm square divided into four quadrants was displayed on the computer screen. During each trial, a character pair consisting of letters, numbers or symbols was presented in the center of one quadrant. Participants had to either perform a letter task in which they classified the letter in the stimulus pair as a consonant or a vowel, or they had to perform a number task in which they classified the number in the pair as odd or even. They were instructed to respond as quickly and accurately as possible for each trial. After their response or after 2,000 ms had passed, a new stimulus pair was displayed in the next quadrant following a clockwise pattern. The upper quadrants were assigned to the letter task and the lower quadrants to the digit task, so that the display location served as a task cue and the task changed predictably. Depending on the task, the relevant character in the stimulus pair was either a letter or a digit, whereas the second and irrelevant character was either a member of the other category, so that the response afforded by this character could be congruent or incongruent with the task-relevant response, or was drawn from a set of neutral characters. This design produced switch trials in Quadrants 1 and 3, and non-switch trials when the stimuli appeared in Quadrants 2 and 4. Consonants were sampled randomly from the set < G, K, M, R >, vowels from the set < A, E, I, U >, even numbers from the set < 2, 4, 6, 8 >, odd numbers from the set < 3, 5, 7, 9 >, and neutral characters from the set < #, ?, ^∗^, % >, with the restriction that a stimulus could not be repeated on successive trials. The position of the task-relevant character within a pair (left or right) was randomly determined on each trial. The participants pressed the left key to indicate “even” or “consonant” and the right key to indicate “odd” or “vowel.” Participants completed a practice set of 9 blocks, each with 16 trials, before entering the experimental phase. This consisted of a set of 15 blocks, with each block again consisting of 16 trials. A short response stimulus interval (RSI) of 150 ms was chosen which remained constant within a given set. A short RSI, the so-called preparation component, has been shown to provoke more pronounced switch costs than long RSI, also known as residual component. This is because shorter intervals usually hamper the reconfiguration process before the stimulus is presented (Colzato et al., 2018a). Stimuli were response-terminated or presented for a maximum duration of 2,000 ms (Figure 2C).

#### Dimensional Change Card Sorting Task

The Dimensional Change Card Sorting task (DCCS) based on Zelazo et al. (2014) was used in the present study to measure set shifting, as recommended by Diamond (2013). This version is part of the NIH Toolbox Cognition Battery and was validated with 268 adults (Zelazo et al., 2014). DCCS makes use of two different styles of bivalent cards, displaying a red rabbit on the left and a blue truck on the right side at the bottom of the screen throughout the task. The participants are then asked to respond to a centrally presented bivalent stimulus (blue/red rabbit/truck) regarding either its shape or color. Pressing the left key sorts the stimulus to the location of the left target (i.e., the red rabbit); pressing the right key sorts the stimulus to the location of the right target (i.e., the blue truck). The DCCS task consists of four blocks (practice, pre-switch, post-switch, and mixed). During the practice block with 24 trials (12 for each dimension), participants receive a feedback whether the response was correct or false. At the beginning of each trial, a fixation cross was shown for 1,000 ms, being followed by the cue (the word “color” or “shape”) they had to respond to. This cue was presented for 1,000 ms. The stimulus was then presented and disappeared only after a response was recorded. Test trials started with a pre-switch block consisting of 15 trials that had the same sorting dimension (color or shape) that was used in the preceding practice block. After that, participants were cued to the other dimension, and a post-switch block with 15 trials took place. When those two blocks are finished, the mixed block begins. Participants are then instructed to sort the stimuli to the dimensions and they are presented with 50 mixed trials that are presented in a pseudorandomized order. This mixed block includes 40 “dominant” and 10 “non-dominant” trials. The dominant dimension, which could be shape or color, was always the sorting dimension that participants were presented to in the post-switch block. The arrangement for all three test blocks is the same as for practice trials, but no feedback is provided. The order of the pre- and post-switch blocks as well as the task version with one of the dominant dimensions was counterbalanced across participants (Figure 2D).

#### Procedure

**FIGURE 3:**
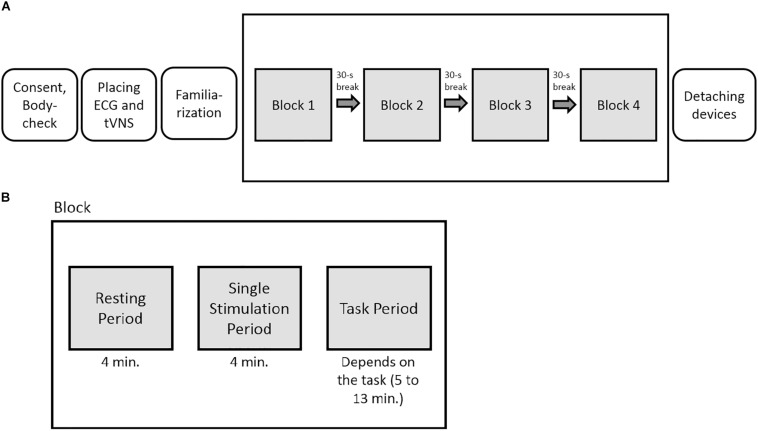
**(A)** Experimental overview. **(B)** Graphical depiction of the phases within each block. In total, the participants underwent four task blocks per testing day in a randomized order.

The experiment had a sham-controlled, single-blinded, randomized crossover within-subject design. For each stimulation condition (active or sham stimulation), the participants underwent all tasks within one session. The order of the tasks was randomized for each participant beforehand. After determining the individual stimulation intensity (familiarization phase), a total of four task blocks were presented, one per task. Each block consisted of one cognitive task and a total of three measurements: The first one was done to take only resting cardiac vagal activity into account (resting period, 4-min measuring interval), the second to measure cardiac vagal activity during the stimulation (tVNS period, 4-min period), and the third to measure cardiac vagal activity during the stimulation simultaneously with the cognitive tasks (task period, 4 min). The tVNS period was included because there is a lack of evidence on the temporal latency of the effects of tVNS (Borges et al., 2019). Thus, a build-up period of four minutes of the effects of tVNS and sham stimulation was used, as done in previous studies (e.g., Burger et al., 2019). Between each test block, the participants could take a 30-s break and were then asked to continue with the next task (Figure 3).

The data collection took place on two different dates with approximately 1 week between the two sessions. During the sessions, either active or sham stimulation was administered to each participant. According to the crossover design, all participants underwent both stimulation conditions. The order of stimulation condition (active-sham; sham-active) was counterbalanced across participants. After taking a seat, signing the informed consent, and answering questions from a body check which included questions related to the exclusion criteria, the ECG and the tVNS electrodes were positioned. The participants then performed the four cognitive tasks across the four blocks. The HRV resting measure was taken in a sitting position with the eyes looking at a gray screen, knees at 90°, and hands on the thighs. The same body position was kept for all measurement periods, and the participants were asked to move as little as possible during the experiment. The order of the tasks was counterbalanced, however, the course of events in both conditions was identical. At the end of the second testing session, the participants were debriefed and thanked.

### Data Analysis

Outliers in the HRV data (less than 1% of the data) were winsorized, meaning that values higher/lower than two standard deviations from the mean were transformed into a value of two standard deviations from the mean. Since the HRV data as well as the tasks data were afterward still positively skewed, they were log-transformed to obtain a normal distribution. We ran the analyses with the log-transformed values; however, we indicate the raw data as descriptive values, given that they can be more easily interpreted. We excluded incorrect and missed responses for all RT analyses, and for all error percentage analyses, incorrect and missed responses were included. We defined the same cut-off values to exclude outliers in the four cognitive tasks, namely responses faster than 200 ms and greater than 2,000 ms.

To test H1a–d, we ran 2 × 2 repeated-measures analyses of variance (rmANOVAs) with stimulation condition (active vs. sham stimulation) and congruency (congruent vs. incongruent trial) for inhibitory control tasks, and stimulation condition (active vs. sham stimulation) and trial type (switch vs. non-switch trial) for cognitive flexibility tasks as within-subject factors. The relevant task parameters are RT and percentage error for all four tasks, and additionally switch costs for the cognitive flexibility tasks. Only for the effect of tVNS on switch costs (RT on switch trials minus RT on repeated trials), paired samples *t*-tests were run. To investigate H2a–d, we ran a 2 (active and sham stimulation) × 3 (resting, single tVNS, and task period) rmANOVA for each task block. Relevant dependent variables were RMSSD, HF, and respiratory frequency. To address H3a–d, we ran separated Pearson product-moment correlation matrices, one for active and one for sham stimulation, for all tasks. We investigated the correlation between RMSSD and HF during the single tVNS period and RT and percentage error, while controlling HF for respiration. In the analysis of the cognitive flexibility tasks, we additionally included switch costs. Finally, to test H4a–d, we did the same analysis as for H3a–d, but considering RMSSD and HF during the tasks instead of during the single tVNS period. To control for false discovery rate (FDR) due to multiple correlation testing, for all correlation matrices we applied the Benjamini–Hochberg procedure which adjust the *p-*value (Benjamini and Hochberg, 1995). For all rmANOVAs, Greenhouse–Geisser correction was used when sphericity was violated. In the case of a significant main or interaction effect, *post hoc* paired sample t-tests with aggregated means were conducted using Bonferroni correction. To quantify evidence for the hypotheses found, we ran Bayesian statistics using Bayesian information criteria (Wagenmakers, 2007) for all analyses. Terms used to discuss the reported Bayes factors are based on Wetzels et al. (2011) recommendations. Accordingly, values higher than 1 provide evidence for alternative hypotheses, whereas values lower than 1 provide evidence for null hypotheses. The Bayes factor can have the following meanings: anecdotal or worth no more than a bare mention (0.333 < *B*_10_ < 3), substantial (0.100 < B_10_ ≤ 0.333 or 3 ≤ *B*_10_ < 10), strong (0.033 < *B*_10_ ≤ 0.100 or 10 < *B*_10_ < 30), very strong (0.010 < *B*_10_ ≤ 0.033 or 30 ≤ *B*_10_ < 100), and decisive (*B*_10_ ≤ 0.010 or *B*_10_ ≥ 100) evidence. To control for carry-over effects on RMSSD and HF, which potentially arose in the current block due to the previous block, we tested the effect of position (i.e., first, second, third and fourth resting periods arranged chronologically) on each testing day. We also took the testing days (Day 1 and Day 2) into account in the same analysis and checked if there was a difference in RMSSD and HF from the first to the second day. We ran two separated 2 (Day 1 and Day 2) × 4 (Resting period 1, Resting period 2, Resting period 3, and Resting period 4) rmANOVAs, one for each vmHRV parameter. Furthermore, we checked whether there was a learning effect in the cognitive tasks from one testing day to the other by running 2 (Day 1 and Day 2) × 2 (congruent and incongruent or non-switch and switch trials, depending on the task) rmANOVAs, one for each behavioral measurement. Finally, to check whether tVNS affects task performance more strongly when its trials are novel, we split the trials of the tasks into first and second half, whereby first half would correspond to novel trials, and collapsed the congruent/non-switch with the incongruent/switch trials. We then ran 2 × 2 rmANOVAs with stimulation (active and sham stimulation) and novelty (first and second half of the task) as factors, and RT and percentage error of all tasks as dependent variables. The results of these additional analyses can be found as a Supplementary Table 1. To report the results of the present study, we followed the CONSORT statement, which stands for Consolidated Standards of Reporting Trials (Dwan et al., 2019). We used IBM SPSS Statistics 26 to prepare the data and JASP 0.11.1 to analyze it. Significance level was α = 0.05.

## Results

### Effects of tVNS on Executive Functions

Descriptive statistics are presented in Table 2, and complete results of the hypothesis testing can be found in Table 3 (inhibitory control tasks) and Table 4 (cognitive flexibility tasks), here we will mainly focus on significant results as well as on results of Bayesian estimations for effects of stimulation. The rmANOVAs revealed that, regarding RTs in the Flanker task, there was an effect of congruency, *F*(1,31) = 95.788, *p* < 0.001, η_*p*_^2^ = 0.755, with RTs in the congruent trials (*M* = 475.93 ms, *SD* = 52.14) being significantly shorter than in the incongruent trials (*M* = 555.38 ms, *SD* = 72.28), *t*(31) = 9.100, *p* < 0.001, *d* = 1.609. No effect of active stimulation compared to sham stimulation could be found *(p* = 0.283). Regarding percentage error in the Flanker task, there was an effect of congruency, *F*(1,31) = 8.202, *p* = 0.007, η_*p*_^2^ = 0.209, with congruent trials (*M* = 4.40%, *SD* = 4.40) presenting less errors than incongruent trials (*M* = 6.80%, *SD* = 7.12), *t*(31) = 3.157, *p* = 0.004, *d* = 0.558. No effect of active stimulation compared to sham stimulation could be found (*p* = 0.760). According to the estimated Bayes factors (alternative/null), data provided substantial evidence for null effects of stimulation condition on RT (*B*_10_ = 0.311) and substantial evidence of null effects in percentage error (*B*_10_ = 0.196).

**TABLE 2:** Mean scores and standard deviations for the performance-relevant parameters of the four cognitive tasks used in the study.

		**RT (ms)**		**Percentage error (%)**		**Switch costs (ms)**	
		**Active Stimulation**	**Sham Stimulation**	**Active Stimulation**	**Sham Stimulation**	**Active Stimulation**	**Sham Stimulation**
Flanker Task	Congruent trials	482.29 (68.19)	469.57 (48.91)	4.50 (4.68)	4.64 (4.45)		
	Incongruent trials	562.54 (88.48)	548.21 (73.63)	7.65 (10.59)	5.95 (5.32)		
Spatial	Congruent trials	501.55 (52.93)	506.60 (60.88)	1.13 (1.40)	1.80 (2.26)		
Stroop Task	Incongruent trials	526.08 (60.86)	537.20 (64.90)	4.45 (3.98)	4.32 (4.24)		
NLT	Non-switch trials	984.11 (164.33)	955.35 (126.00)	21.96 (4.25)	23.41 (2.44)		
	Switch trials	1,212.09(148.21)	1,205.95(141.31)	20.12 (4.20)	20.65 (3.85)		
						225.23 (107.14)	251.08 (97.47)
DCCS	Non-switch Trials	600.16 (138.69)	577.51 (113.56)	18.31 (16.01)	16.68 (15.48)		
	Switch Trials	603.90 (137.04)	614.01 (138.65)	28.24 (23.92)	27.76 (24.32)		
						4.77 (39.75)	37.54 (45.39)

*RT, reaction times; NLT, Number Letter task; DCCS, Dimensional Card Sorting task.*

**TABLE 3:** Inhibitory control tasks: results of repeated measures analyses of variance for the performance-related as well as heart rate variability parameters, with Bayesian analyses (*B*_10_).

**Flanker task**					**Spatial Stroop task**			
	***F*-value**	***p*-value**	**η _*p*_^2^**	***B*_10_**	***F*-value**	***p*-value**	**η _*p*_^2^**	***B*_10_**
**RT**								
Congruency	95.788	< 0.001	0.755	2.018E+13	39.001	< 0.001	0.557	2,732.297
Stimulation condition	1.192	0.283		0.311	0.860	0.361		0.344
Stimulation × congruency	0.001	0.992		0.280	0.754	0.392		3.047
**Percentage error**								
Congruency	8.202	0.007	0.209	3.796	37.673	< 0.001	0.549	4.204E+7
Stimulation condition	0.095	0.760		0.196	0.098	0.756		0.201
Stimulation × congruency	0.511	0.480		0.278	2.626	0.115		0.596
**RMSSD**								
Stimulation condition	0.250	0.621		0.215	0.009	0.926		0.189
Time measurements	2,862	0.065		0.220	2.576	0.084		0.154
Time × condition	0.351	0.645		0.048	3.845	0.027	0.110	0.372
**HF**								
Stimulation condition	1.669	0.211		0.664	0.012	0.915		0.196
Time measurements	2.291	0.135		0.632	2.146	0.132		0.726
Time × condition	3.038	0.059		0.158	0.681	0.512		0.203
**Respiratory frequency**								
Stimulation condition	0.714	0.405		0.617	0.213	0.648		0.227
Time measurements	3.518	0.047	0.102	0.102	2.917	0.062		0.099
Time × condition	0.855	0.430		0.010	0.109	0.897		0.087

*RT, reaction times; RMSSD, root mean square of the successive differences; HF, high frequency.*

**TABLE 4:** Cognitive flexibility tasks: results of repeated measures analyses of variance for the performance-related as well as heart rate variability parameters, with Bayesian analyses (*B*_10_).

	**NLT**				**DCCS**			
	***F*-value**	***p*-value**	**η_*p*_^2^**	***B*_10_**	***F*-value**	***p*-value**	**η_*p*_^2^**	***B*_10_**
**RT**								
Trial type	225.365	< 0.001	0.879	1.446E+22	14.720	0.001	0.322	0.314
Stimulation condition	0.454	0.505		0.210	0.015	0.904		0.192
Stimulation x congruency	1.670	0.206		0.411	11.106	0.002	0.264	0.339
**Percentage error**								
Trial type	59.615	< 0.001	0.658	602.764	15.343	< 0.001	0.331	0.491
Stimulation condition	1.996	0.168		1.097	0.177	0.677		0.233
Stimulation × congruency	3.214	0.083		0.382	0.552	0.463		0.250
**Switch costs^1^**	1.513	0.140		0.529	2.797	0.009	0.494	4.916
**RMSSD**								
Stimulation condition	< 0.001	0.991		0.152	0.024	0.877		0.160
Time measurements	0.517	0.599		0.073	1.590	0.212		0.133
Time × condition	0.810	0.449		0.011	1.269	0.288		0.150
**HF**								
Stimulation condition	0.324	0.575		0.216	0.217	0.646		0.186
Time measurements	4.689	0.014	0.039	12.853	6.821	0.002	0.078	260.327
Time × condition	1.061	0.355		0.163	0.391	0.679		0.130
**Respiratory frequency**								
Stimulation condition	0.021	0.885		0.159	0.010	0.920		0.168
Time measurements	0.657	0.522		0.078	1.516	0.228		0.078
Time x condition	0.508	0.604		0.100	0.545	0.582		0.083

*^1^The depicted results are from *t*-tests. Consequently, for switch costs, instead of *F* and η_*p*_^2^, the results are for *t*-values and Cohen’s *d*, respectively. NLT, Number Letter task; DCCS, Dimensional Card Sorting test; RT, reaction times; RMSSD, root mean square of the successive differences; HF, high frequency.*

For RT in the Spatial Stroop task, there was an effect of congruency, *F*(1,31) = 39.001, *p* < 0.001, η_*p*_^2^ = 0.557, with RTs in the congruent trials (*M* = 504.08 ms, *SD* = 51.73) being significantly shorter than in the incongruent trials (*M* = 531.64 ms, *SD* = 56.21), *t*(31) = 6.245, *p* < 0.001, *d* = 1.104. No effect of active stimulation compared to sham stimulation could be found *(p* = 0.361). Regarding percentage error, there was an effect of congruency, *F*(1,31) = 37.673, *p* < 0.001, η_*p*_^2^ = 0.549, with congruent trials (*M* = 1.47%, *SD* = 1.48) presenting less errors than incongruent trials (*M* = 4.39%, *SD* = 3.63), *t*(31) = 6.138, *p* < 0.001, *d* = 1.085. No effect of active stimulation compared to sham stimulation could be found (*p* = 0.756). According to the estimated Bayes factors, data provided anecdotal evidence against the alternative hypothesis for stimulation condition regarding RT (*B*_10_ = 0.344) and substantial evidence against evidence for effects of stimulation on percentage error (*B*_10_ = 0.201). Furthermore, Bayesian estimation indicated substantial evidence for an interaction effect (*B*_10_ = 3.047).

For NLT, an effect of trial type (switch trial vs. non-switch trial) could be found on RT, *F*(1,31) = 225.365, *p* < 0.001, η_*p*_^2^ = 0.879, with non-switch trials (*M* = 969.73 ms, *SD* = 130.41) having shorter RT than switch trials (*M* = 1,209.02 ms, *SD* = 127.84), *t*(31) = 15.012, *p* < 0.001, *d* = 2.654. No effect of active stimulation compared to sham stimulation could be found regarding RT (*p* = 0.505). Switch costs during active stimulation (*M* = 225.23 ms, *SD* = 107.14) and during sham stimulation (*M* = 251.08 ms, *SD* = 97.47) did not differ from each other, *p* = 0.140. Regarding percentage error, there was an effect of trial type, *F*(1,31) = 59.615, *p* < 0.001, η_*p*_^2^ = 0.658, with non-switch trials (*M* = 22.68%, *SD* = 2.91) presenting more errors than switch trials (*M* = 20.39%, *SD* = 3.22), *t*(31) = 7.721, *p* < 0.001, *d* = 1.365. There was no main effect of stimulation (*p* = 0.168). Bayes factor indicates substantial evidence against the alternative hypothesis for stimulation condition regarding RT (*B*_10_ = 0.210), anecdotal evidence supporting the effect of stimulation on percentage error (*B*_10_ = 1.097), and anecdotal evidence against the effect of tVNS on switch costs (*B*_10_ = 0.529).

For DCCS, an effect of trial type on RT could be found, *F*(1,31) = 14.720, *p* = 0.001, η_*p*_^2^ = 0.322, with non-switch trials (*M* = 969.73 ms, *SD* = 130.41) having shorter RT than switch trials (*M* = 1,209.02 ms, *SD* = 127.84), *t*(31) = 15.012, *p* < 0.001, *d* = 2.654. There was no effect of stimulation on RT (*p* = 0.904), but there was an interaction effect between trial type and stimulation conditions, *F*(1,31) = 11.106, *p* = 0.002, η_*p*_^2^ = 0.264. *Post hoc* analyses (Bonferroni-corrected *p* = 0.0125) revealed that RT in non-switch trials during the sham stimulation condition (*M* = 557.51 ms, *SD* = 113.56) was significantly lower than RT in switch trials during the sham condition (*M* = 614.01 ms, *SD* = 138.65), *t*(31) = 4.767, *p* < 0.001, *d* = 0.843. Regarding percentage error, there was an effect of trial type, *F*(1,31) = 15.343, *p* < 0.001, η_*p*_^2^ = 0.331, with non-switch trials having a lower percentage error (*M* = 17.49%, *SD* = 11.39) than switch trials (*M* = 28.00%, *SD* = 17.30), *t*(31) = 3.917, *p* < 0.001, *d* = 0.692. There was no effect of stimulation on RT (*p* = 0.677). Active and sham stimulation differed significantly regarding switch costs, with switch costs during active stimulation (*M* = 4.77 ms, *SD* = 39.75) being lower than during sham condition (*M* = 37.54 ms, *SD* = 45.39), *t*(31) = 2.797, *p* = 0.009, *d* = 0.494. Bayes factor indicates substantial evidence against any effects of stimulation condition on RT (*B*_10_ = 0.192), against the alternative hypothesis for percentage error (*B*_10_ = 0.233), and substantial evidence for the differences in switch costs (*B*_10_ = 4.916). Furthermore, Bayesian estimation indicated substantial evidence for an interaction effect (*B*_10_ = 3.047).

### Effects of tVNS on Cardiac Vagal Activity

Descriptive statistics are presented in Table 5, and complete results of the hypothesis testing can be found in Table 3 (inhibitory control tasks) and Table 4 (cognitive flexibility tasks), here we will mainly focus on significant results as well as on results of Bayesian estimations for effects of stimulation. Regarding changes of cardiac vagal activity within the test blocks (i.e., between resting, single tVNS, and tVNS with task periods, as well as between active and sham stimulation), for Flanker task there was neither a main effect of stimulation condition (*p* = 0.621), nor of time on RMSSD (*p* = 0.065). The same applies to the main effects on HF (stimulation condition: *p* = 0.135; time: *p* = 0.221). There was no effect of stimulation on respiratory frequency (*p* = 0.405), but an effect of time, *F*(1.587,49.206) = 3.518, *p* = 0.047, η_*p*_^2^ = 0.102. However, *post hoc* analyses (Bonferroni-corrected *p* = 0.017) revealed no significant mean differences. According to the estimated Bayes factors, data provided substantial evidence against the alternative hypothesis for stimulation condition regarding RMSSD (*B*_10_ = 0.215), and anecdotal evidence regarding HF (*B*_10_ = 0.664).

**TABLE 5:** Mean scores and standard deviations for the heart rate variability parameters over time in the four cognitive task blocks.

		**RMSSD (ms)**		**HF (ms^2^)**		**Respiratory frequency (cycles per minute)**	
		**Active Stimulation**	**Sham Stimulation**	**Active Stimulation**	**Sham Stimulation**	**Active Stimulation**	**Sham Stimulation**
Flanker Task	Resting	48.43 (22.38)	52.34 (26.56)	13.81 (8.78)	13.71 (12.45)	12.36 (2.06)	12.19 (2.68)
	tVNS	52.56 (28.53)	54.66 (25.02)	15.27 (11.26)	19.59 (13.94)	12.51 (2.40)	12.10 (2.91)
	Task	55.44 (29.81)	55.26 (24.66)	14.44 (9.60)	16.16 (11.56)	12.23 (2.33)	11.66(3.03)
Spatial	Resting	52.38 (27.64)	53.48 (21.52)	12.97 (10.05)	14.12 (10.80)	14.70 (9.61)	15.85 (10.79)
Stroop Task	tVNS	54.47(25.99)	58.85 (26.31)	18.74(13.19)	17.31(13.60)	19.60(11.82)	19.16(15.58)
	Task	55.93(26.89)	50.70(19.28)	15.65 (8.45)	17.45 (13.91)	16.25 (9.12)	20.32 (16.48)
NLT	Resting	51.82(24.75)	50.07 (22.2)	18.06(12.22)	13.83(10.98)	12.20 (2.03)	12.02(2.33)
	tVNS	49.91(21.12)	51.82(20.44)	18.51(12.56)	18.85 (15.07)	12.27 (2.05)	12.38(2.64)
	Task	50.28(25.77)	48.78(18.45)	17.78(12.13)	17.547(9.40)	12.06 (1.88)	12.17(2.48)
DCCS	Resting	54.26(24.46)	51.82(22.46)	14.93(10.11)	16.24(14.98)	13.52 (8.82)	15.66(13.96)
	tVNS	54.90(25.86)	57.4 (24.75)	17.56(12.57)	19.59(13.11)	17.95(12.18)	19.23(12.80)
	Task	56.36(24.52)	55.41(23.24)	19.83(13.16)	17.55(11.14)	20.76(11.75)	19.90(10.07)

*RMSSD, root mean square of the successive differences; HF, high frequency; tVNS, transcutaneous vagus nerve stimulation (single stimulation phase); NLT, Number Letter task; DCCS, Dimensional Change Card Sorting task.*

For the Spatial Stroop task, neither a main effect of stimulation on RMSSD (*p* = 0.926), nor of time (*p* = 0.084), was found. There was an interaction effect between the stimulation condition and RMSSD, *F*(2,62) = 3.845, *p* = 0.027, η_*p*_^2^ = 0.110, however, *post hoc* analyses revealed no effects after Bonferroni correction (*p* = 0.006). There was no effect of stimulation (*p* = 0.915), and time (*p* = 0.132) on HF and no effects on respiratory frequency (stimulation: *p* = 0.648, time: *p* = 0.062). Bayes factor indicates substantial evidence against the alternative hypothesis for stimulation condition regarding RMSSD (*B*_10_ = 0.189), HF (*B*_10_ = 0.196), and respiratory frequency (*B*_10_ = 0.227).

For the NLT, there was neither an effect of stimulation on RMSSD (*p* = 0.991), nor on time (*p* = 0.599). Regarding HF, no effect of stimulation (*p* = 0.575), but a main effect of time was found, *F*(2,46) = 4.689, *p* = 0.014, η_*p*_^2^ = 0.039. *Post hoc* analyses (Bonferroni-corrected *p* = 0.017) revealed that HF during the resting period (*M* = 12.92, *SD* = 8.25) was significantly lower than during the task period (*M* = 18.31, *SD* = 9.39), *t*(31) = 4.108, *p* < 0.001, *d* = 0.726. According to the estimated Bayes factors, there is substantial evidence against the alternative hypothesis for stimulation condition regarding RMSSD (*B*_10_ = 0.152), regarding HF (*B*_10_ = 0.216), and respiratory frequency (*B*_10_ = 0.159).

For the DCCS, there was neither a main effect of stimulation condition on RMSSD (*p* = 0.877), nor of time (*p* = 0.212). Regarding HF, there was no effect of stimulation, (*p* = 0.646), but a main effect of time, *F*(1.613,38.708) = 6.821, *p* = 0.002 η_*p*_^2^ = 0.078. *Post hoc* analyses (Bonferroni-corrected *p* = 0.017) revealed that HF increased from resting (*M* = 13.36, *SD* = 9.42) to single stimulation phase (*M* = 16.71, *SD* = 11.20), *t*(31) = 3.205, *p* = 0.003, *d* = 0.566, and from resting to task phase (*M* = 19.71, *SD* = 8.96), *t*(31) = 4.708, *p* < 0.001, *d* = 0.832. According to the estimated Bayes factors, data provided substantial evidence against the alternative hypothesis for RMSSD regarding stimulation condition (*B*_10_ = 0.160), regarding HF (*B*_10_ = 0.186), and regarding respiratory frequency (*B*_10_ = 0.168).

### Correlations Between Cardiac Vagal Activity and Cognitive Performance

We ran Pearson product-moment correlations to investigate if vmHRV parameters that were measured during the single stimulation phase and the task phase predicted performance on the cognitive tasks. Complete correlation matrices can be found in Table 6 (for inhibitory control tasks) and Table 7 (for cognitive flexibility tasks), here we will only present significant results. None of the vmHRV parameters measured during the Flanker task correlated with the cognitive parameters. Regarding the Spatial Stroop task, there was only significant correlations between the parameters measured in the sham condition: RT in both congruent (*r* = −0.42, *p* = 0.018) and incongruent trials (*r* = −0.39, *p* = 0.027) correlated negatively with RMSSD during the single stimulation phase. HF correlated negatively with RT in the congruent trials during the single stimulation phase (*r* = −0.43, *p* = 0.038), and positively with percentage error of the incongruent trials during the single stimulation phase (*r* = 0.43, *p* = 0.032). In the NLT, RMSSD correlated positively with percentage error of non-switch trials during the active condition (*r* = 0.40, *p* = 0.025). In the active condition, HF during the single stimulation phase correlated negatively with RT of both non-switch (*r* = −0.44, *p* = 0.015) and switch trials (*r* = −0.50, *p* = 0.005), and HF during the task phase correlated negatively with switch costs (*r* = −0.42, *p* = 0.019). In the sham condition, HF correlated positively with percentage error during the task phase (*r* = 0.48, *p* = 0.015). In the DCCS, switch costs in the active condition correlated positively with RMSSD during the single stimulation phase (*r* = 0.40, *p* = 0.024), with RMSSD during the task phase (*r* = 0.37, *p* = 0.035), and negatively with HF during the task phase (*r* = −0.42, *p* = 0.019). HF during the task phase correlated positively with RT of both non-switch (*r* = −0.40, *p* = 0.026) and switch trials (*r* = −0.42, *p* = 0.018). Importantly, after adjusting the *p*-values using the FDR correction, none of these correlations remained significant.

**TABLE 6:** Pearson product-moment correlations between cognitive performance-relevant parameters and vagally-mediated heart rate variable parameters during the single stimulation phase (tVNS) and the task phase (task) for active and sham conditions.

			**Active Stimulation**				**Sham Stimulation**			
			**RT**		**Percentage error**		**RT**		**Percentage error**	
			**Congruent trials**	**Incongruent trials**	**Congruent trials**	**Incongruent trials**	**Congruent trials**	**Incongruent trials**	**Congruent trials**	**Incongruent trials**
**Flanker task**										
RMSSD	tVNS	Pearson’s *r*	0.02	–0.05	–0.21	–0.22	–0.29	–0.23	0.26	0.197
		*p*-value	0.935	0.768	0.243	0.233	0.114	0.207	0.144	0.280
	Task	Pearson’s *r*	–0.06	–0.08	–0.25	–0.28	–0.24	–0.24	0.16	0.27
		*p*-value	0.760	0.666	0.171	0.115	0.193	0.189	0.369	0.140
HF	tVNS	Pearson’s *r*	–0.20	–0.19	0.16	0.23	–0.24	0.01	0.06	0.03
		*p*-value	0.288	0.334	0.395	0.237	0.262	0.991	0.785	0.906
	Task	Pearson’s *r*	–0.25	–0.30	–0.09	–0.17	–0.34	–0.18	–0.01	0.07
		*p*-value	0.189	0.119	0.647	0.378	0.109	0.394	0.987	0.760
**Spatial Stroop task**										
RMSSD	tVNS	Pearson’s *r*	0.06	–0.04	–0.22	–0.15	−0.42*	−0.39*	–0.12	–0.01
		*p*-value	0.755	0.845	0.227	0.403	0.018	0.027	0.532	0.987
	Task	Pearson’s *r*	–0.02	–0.13	–0.18	–0.34	–0.34	–0.31	0.19	0.12
		*p*-value	0.907	0.485	0.318	0.054	0.059	0.088	0.311	0.514
HF	tVNS	Pearson’s *r*	–0.25	–0.27	–0.11	0.28	−0.43*	–0.32	0.17	0.45*
		*p*-value	0.175	0.151	0.579	0.131	0.038	0.142	0.437	0.032
	Task	Pearson’s *r*	–0.20	–0.07	–0.17	0.12	–0.27	–0.24	0.11	0.10
		*p*-value	0.302	0.715	0.376	0.539	0.219	0.264	0.623	0.642

Coefficients for the inhibitory control tasks. *p < 0.05. Non-adjusted *p-*values. RT, reaction times; RMSSD, root mean square of successive differences; HF, high frequency; tVNS, transcutaneous vagus nerve stimulation (single stimulation phase).

**TABLE 7:** Pearson product-moment correlations between cognitive performance-relevant parameters and vagally mediated heart rate variable parameters during the single stimulation phase (tVNS) and the task phase (task) for active and sham conditions. Coefficients for the cognitive flexibility tasks.

			**Active Stimulation**					**Sham Stimulation**				
			**RT**		**Percentage error**			**RT**		**Percentage error**		
			**Non-switch trials**	**Switch trials**	**Non-switch trials**	**Switch trials**	**Switch costs**	**Non-switch trials**	**Switch trials**	**Non-switch trials**	**Switch trials**	**Switch costs**
**NLT**												
RMSSD	tVNS	Pearson’s *r*	–0.28	–0.24	0.40*	0.14	0.13	0.12	0.15	0.19	–0.06	–0.02
		*p*-value	0.132	0.179	0.025	0.434	0.434	0.513	0.430	0.308	0.727	0.934
	Task	Pearson’s *r*	–0.06	–0.03	0.33	0.31	0.13	0.10	0.29	0.22	–0.02	0.28
		*p*-value	0.732	0.860	0.070	0.081	0.475	0.595	0.113	0.238	0.909	0.115
HF	tVNS	Pearson’s *r*	−0.44*	−0.50**	0.37*	0.31	0.14	0.09	–0.10	0.11	–0.23	–0.28
		*p*-value	0.015	0.005	0.046	0.099	0.463	0.677	0.626	0.599	0.279	0.170
	Task	Pearson’s *r*	−0.39*	–0.24	0.28	0.22	0.42*	–0.10	–0.02	0.48*	0.07	0.15
		*p*-value	0.034	0.204	0.129	0.242	0.020	0.621	0.914	0.015	0.748	0.482
**DCCS**												
RMSSD	tVNS	Pearson’s *r*	–0.27	–0.17	0.24	0.29	0.40*	0.09	0.04	–0.03	–0.01	–0.10
		*p*-value	0.134	0.351	0.180	0.103	0.024	0.623	0.837	0.869	0.973	0.603
	Task	Pearson’s *r*	–0.25	–0.14	0.16	0.23	0.37*	0.06	0.01	0.03	0.02	–0.08
		*p*-value	0.177	0.440	0.385	0.212	0.035	0.741	0.953	0.858	0.920	0.660
HF	tVNS	Pearson’s *r*	−0.40*	−0.42*	0.29	0.31	–0.08	0.05	0.04	–0.19	–0.14	0.03
		*p*-value	0.026	0.018	0.110	0.089	0.684	0.796	0.835	0.356	0.483	0.900
	Task	Pearson’s *r*	0.07	–0.01	–0.27	–0.19	−0.42*	–0.05	–0.03	0.02	–0.06	0.10
		*p*-value	0.715	0.970	0.150	0.314	0.019	0.819	0.905	0.931	0.761	0.619

*p < 0.05, **p < 0.01. Non-adjusted *p* values. RT = reaction times; RMSSD = root mean square of successive differences; HF = high frequency; NLT = Number Letter task; DCCS = Dimensional Change Card Sorting task; tVNS = transcutaneous vagus nerve stimulation (single stimulation phase).

## Discussion

The aim of this study was to investigate the effect of tVNS on performance in tasks commonly used to measure inhibitory control and cognitive flexibility, core executive functions on which higher-order executive functions rely. Based on the neurovisceral integration model (Thayer et al., 2009), we hypothesized that executive performance would be better during the active stimulation condition compared to the sham stimulation condition (H1a–d). Conflict effects were found in all four tasks used. However, among the four tasks, only in the DCCS a better performance could be directly linked to tVNS, with switch costs being lower in the active condition than in the sham condition. For this reason, among the H1 hypotheses, only H1c was supported. On the physiological level, we expected vmHRV to be higher in the active condition during both the single stimulation period and the task period (H2a–d). During both cognitive flexibility tasks, HF increased from resting phase to task phase, but no difference between active and sham stimulation could be detected. Therefore, H2a–d were not supported. Moreover, it was hypothesized that higher cardiac vagal activity in the single stimulation phase (H3a–d) and in the task phase (H4a–d) would be associated with better task performance only in the active condition. Because none of the correlations remained significant after adjusting the *p*-values, none of these hypotheses could be confirmed.

In the present study, we could provide a conceptual replication (Walker et al., 2017) of the conflict effects previously observed in tasks that are thought to mainly demand selective attention like the Flanker task (Alderman and Olson, 2014) and response inhibition with the Spatial Stroop task (Marotta et al., 2018). In the same sense, findings toward dual-task interference evoked by a task used to measure task switching with NLT (Colzato et al., 2018a), as well as by a task thought to measure set shifting with DCCS (Zelazo et al., 2014) could be replicated with large effect sizes. However, an effect of tVNS could be found only on set shifting with DCCS. First, smaller switch costs during tVNS were observed compared to the sham condition. Second, RT in non-switch trials did not differ from RT in switch trials during active stimulation, but in the sham stimulation RT in switch trials were higher than in non-switch trials. Possibly tVNS diminished the dual-task interference, whereas sham stimulation did not, and this would explain this difference in switch costs between tVNS and sham stimulation. Importantly, some results referring to a lack of difference between active and sham stimulation were not substantially supported by Bayesian estimations, namely for RT in the Spatial Stroop task, HF and respiratory frequency in the Flanker task, and percentage error and switch costs in the NLT. Consequently, these findings should be interpreted carefully.

The mixed nature of the results and the lack of correlation between cognitive performance and cardiac vagal activity provide evidence against a generability of the neurovisceral integration model (Thayer et al., 2009). These findings can be interpreted in various manners. First, the present study indicates that tVNS may exert a circumscribed influence on core executive functions. This suggests that the neurovisceral integration model may be less generally applicable than previously outlined (Thayer et al., 2009; Smith et al., 2017). This specificity is in line with previous findings involving executive functions and cardiac vagal activity (Jennings et al., 2015). Jennings et al. (2015) found that cardiac vagal activity was not directly related to resting state activity of intrinsic brain networks but rather to more localized connectivity. This implies that the integration between autonomic and cognitive control is more limited than the general integration originally suggested. Consequently, the neurovisceral integration model (Thayer et al., 2009) might not apply to the full range of executive functions, but rather to specific cognitive functions (Jennings et al., 2015).

It is not clear, however, whether the specificity of the integration between autonomic and cognitive regulation shown in the present study is valid for executive functions in general–i.e., independently of the method used to manipulate them–or whether tVNS affects only specific cognitive regulation processes. One of the reasons for this possible specificity related to tVNS might lie in the level of neurotransmission: tVNS sends a signal to the locus coeruleus (Kraus et al., 2007; Dietrich et al., 2008), the primary source of norepinephrine in the brain (Foote et al., 1983). Norepinephrine has been thought to be engaged by tVNS (Steenbergen et al., 2015; van Leusden et al., 2015; Beste et al., 2016). Locus coeruleus plays an important role in reorienting attention and cognitive flexibility, and those neurons have been shown to have a task-related activation (Sara, 2015). Noradrenergic α-1 and α-2 receptors act in distinct cognitive processes: whereas α-2 receptors engage at moderate rates of norepinephrine release, thus promoting working memory, α-1 receptors are activated at higher rates, promoting both focused and flexible attention (Berridge and Spencer, 2016). It is not clear whether DCCS demands more flexible attention than NLT, and whether the difference between the two could only be observed because tVNS evokes a stronger release of norepinephrine, engaging α-1 receptors that were necessary for the DCCS but less so for the NLT. Hence, it is recommended for future studies to address the possible specific efficacy of tVNS by considering an on-line measurement of norepinephrine such as pupillary responses (Warren et al., 2018; Keute et al., 2019b; Burger et al., 2020). This approach might complement and further specify the hypotheses based on the neurovisceral integration model (Thayer et al., 2009).

Second, despite all efforts in taking well-acknowledged recommendations into account, task impurity (Miyake et al., 2000) may not have been ruled out. Consequently, the question remains whether other cognitive processes underlying the specific task used to measure set shifting, and not set shifting *per se*, are influenced by tVNS. For instance, inhibitory processes have been thought to take place in cognitive flexibility. Accordingly, for the efficient activation of another set in the context of set shifting, the inhibition of the previous, no longer relevant task, is required. Therefore, backward inhibition is a process highly involved in cognitive flexibility (Mayr and Keele, 2000). It remains unclear if a comparable amount of backward inhibition is required for both tasks used to measure cognitive flexibility. Similarly, rather than Spatial Stroop task being considered a good index of response inhibition, possibly interference control, i.e., control at the level of perception, is measured by means of this task (Tafuro et al., 2019). To overcome these concerns, it is necessary to develop cognitive tasks that minimally vary from one another in the sense that the additional cognitive processes necessary for performing a cognitive task can be minimized or at least kept constant. This would enable a more accurate integrative assessment of the core executive functions in future research with tVNS investigating executive performance.

Third, the lack of a difference between tVNS and sham stimulation regarding cardiac vagal activity, which is in line with previous findings (Burger et al., 2016, 2019; De Couck et al., 2017; Borges et al., 2019), could have contributed to the heterogeneity of the findings. Despite ample evidence on the effects of tVNS on cognition (e.g., Steenbergen et al., 2015; Sellaro et al., 2017), the evidence provided by the present study on cardiac vagal activity substantiates the arguments against the suitability of the earlobe as a sham stimulation, as discussed lately (Keute et al., 2018b; Rangon, 2018; Borges et al., 2019). At present, there is only one detailed description of the nerve distribution of the human auricle and it shows that the earlobe is free from vagal innervation (Peuker and Filler, 2002). However, it lacks substantial evidence that electrical stimulation on the earlobe cannot stimulate brain center nuclei that trigger an increase in cardiac vagal outflow (Rangon, 2018). This is especially relevant because the boundaries between particular dermatomes often overlap (Butt et al., 2019), so that a clear understanding of the nerve distribution of the human auricle is needed. Regardless of the suitability of the earlobe, it has also been discussed whether vmHRV parameters are sensitive to afferent vagal changes triggered by tVNS; it is not yet clear whether the electrical signal produced by tVNS is strong enough to overcome body-related barriers such as skin and blood vessels, and therefore to trigger vagal afferent firing in a way that would robustly increase prefrontal activity, thus indirectly affecting cardiac vagal activity (Borges et al., 2019).

In the present study, the cognitive tasks themselves did not seem to have an impact on the vmHRV parameters, since neither RMSSD nor HF decreased during the tasks when compared to before the tasks. It is not clear whether this lack of a decrease–which would be expected based on the neurovisceral integration model (Thayer et al., 2009; Smith et al., 2017), given the conflict effects elicited by the tasks–was due to tVNS or not. Possibly, the tasks were not cognitively demanding enough to evoke a decrease in cardiac vagal activity. The lack of cognitive demand could also explain why we found no effect of tVNS on inhibitory control, whereas an array of previous studies provided evidence in this direction (see Table 1). Importantly, none of these previous studies used the same paradigms that were used in the present study. It is possible that the paradigms for measuring inhibitory control used here, at least concerning the amount of trials and instructions used in the present study, are not sensitive to effects that might otherwise be elicited by tVNS. Moreover, none of the previous studies investigating the effects of tVNS on inhibitory control found overall enhanced performance, measured by means of RT and percentage error (see Table 1). Instead, they addressed inhibitory control in specific contexts, such as backward inhibition when working memory is more strongly demanded (Beste et al., 2016), or response selection during action cascading (Steenbergen et al., 2015). Regarding cognitive demand, future studies should incorporate measures of the cognitive demand of the tasks, for instance by means of subjective questionnaires or imaging techniques such as functional near-infrared spectroscopy (fNIRS) and fMRI to measure prefrontal activity during task performance.

As the only vmHRV parameter to show changes in the present study, HF increased during the NLT and DCCS when compared to the resting phase. Since both tasks are cognitively demanding due to the dual-task interference, based on the neurovisceral integration model (Thayer et al., 2009) HF should decrease compared to both resting and single stimulation phases. At the same time, this increase of HF was not associated with a better performance in the DCCS, as it would be predicted by the neurovisceral integration model. Although there was no difference between tVNS and sham stimulation regarding HF in the present study, the increase in HF during the DCCS might be linked to the positive effect of tVNS found on switch costs. So far, there has been no other study investigating the effect of tVNS on respiration, and whether respiration, when affected by tVNS, moderates executive performance. Future studies should address this question in order to further investigate the mechanisms of action behind tVNS.

### Limitations

There are limitations to our study that should be mentioned. First, RMSSD increased within the experimental sessions (see Supplementary Material). It is not clear, however, whether this carry-over effect emerged from the stimulation itself, or simply from the fact that the participants were sitting during the experiment. Thus, this increase during the experimental sessions may represent a relevant confounder that renders it difficult to interpret cardiac vagal activity measurements. Second, despite considering inhibitory control and cognitive flexibility differentially by taking different aspects into account, the present study did not consider other types of cognitive flexibility. Creatively thinking outside the box, seeing something from different perspectives (Diamond, 2013), or stochastic reversal learning (Colzato et al., 2018a) could be aspects of cognitive flexibility prone to be influenced by tVNS. Third, respiratory frequency was obtained via a dedicated algorithm from Kubios (Tarvainen et al., 2013). However, a more precise assessment of respiratory frequency such as a respiration belt or a pneumotachograph is recommendable (Quintana et al., 2016). Fourth, the sample has a misbalance regarding gender, with male participants being vast majority. Given that sex differences can influence cardiac vagal activity (Koenig and Thayer, 2016), this misbalance may have been an issue for the analysis. Finally, as stated above, the tasks are not comparable to each other. For example, the Flanker task used here has, when compared to the Spatial Stroop task, a shorter stimulus presentation time and random intertrial interval. This may provoke different cognitive processes that deviate from the ones we aimed at measuring. A further difference is the length of the tasks, ranging from five (DCCS) to 13 (Flanker task) min. The amount of trials also greatly varies between the tasks. Due to a lack of measurement of task difficulty, it was not possible to investigate whether the difficulty level differed strongly between the tasks, as stated above. Furthermore, the DCCS uses colorful pictures, whereas all other tasks are bicolored and involve time pressure. The impact of these differences on the cognitive tasks should be considered when using them in future studies with tVNS.

## Conclusion

The present study is the first to investigate different core executive functions with their different subtypes in an integrative manner. Additionally, this is the first study to investigate the effect of tVNS on cognitive flexibility. On the one hand, it was shown that tVNS can lead to less switch costs in set shifting, possibly explained by diminished dual-task interference due to tVNS. On the other hand, the present study provided evidence that tVNS may have only very specific effects on cognitive processes. By addressing the different aspects of core cognitive functions in one standardized study design, the present study contributes to a better understanding of the effects of tVNS by further delineating what kind of cognitive and physiological mechanisms might be influenced by this neuroenhancement tool. Future studies investigating the effect of tVNS on executive functions should further investigate cognitive flexibility and consider task characteristics as well as address different types of executive functions.

## Data Availability Statement

All datasets generated for this study are included in the article/Supplementary Material.

## Ethics Statement

This study was reviewed and approved by the Ethics Committee of the German Sport University Cologne (120/2018). The participants provided their written informed consent to participate in this study.

## Author Contributions

UB and SL contributed to conceiving the design of the study. LK led the data collection with the help of UB. UB realized the statistical analysis with the help of SL. UB wrote the first draft of the manuscript. SL, MR, and SK provided critical comments to improve it. SL and MR suggested the final adjustments on the manuscript. All authors agreed on the final version.

## Conflict of Interest

The authors declare that the research was conducted in the absence of any commercial or financial relationships that could be construed as a potential conflict of interest.

## References

[B1] Aihara M. Ida I. Yuuki N. Oshima A. Kumano H. Takahashi K. (2007). HPA axis dysfunction in unmedicated major depressive disorder and its normalization by pharmacotherapy correlates with alteration of neural activity in prefrontal cortex and limbic/paralimbic regions. *Psychiatry Res. Neuroimaging* 155 245–256. 10.1016/j.pscychresns.2006.11.002 17587554

[B2] Alderman B. L. Olson R. L. (2014). The relation of aerobic fitness to cognitive control and heart rate variability: a neurovisceral integration study. *Biol. Psychol.* 99 26–33. 10.1016/j.biopsycho.2014.02.007 24560874

[B3] Arnsten A. F. T. Li B. (2004). Neurobiology of executive functions?: catecholamine influences on prefrontal cortical functions. *Biol. Psychiatry* 57 1377–1384. 10.1016/j.bps.2004.08.019 15950011

[B4] Badran B. W. Dowdle L. T. Mithoefer O. J. LaBate N. T. Coatsworth J. Brown J. C. (2018). Neurophysiologic effects of transcutaneous auricular vagus nerve stimulation (taVNS) via electrical stimulation of the tragus: a concurrent taVNS/fMRI study and review. *Brain Stimul.* 11 492–500. 10.1016/j.brs.2017.12.009 29361441 PMC6487660

[B5] Benjamini Y. Hochberg Y. (1995). Controlling the false discovery rate: a practical and powerful approach to multiple testing. *J. R. Stat. Soc. Ser. B* 57 289–300. 10.1111/j.2517-6161.1995.tb02031.x

[B6] Berridge C. W. Spencer R. C. (2016). Differential cognitive actions of norepinephrine a2 and a1 receptor signaling in the prefrontal cortex. *Brain Res.* 1641 189–196. 10.1016/j.brainres.2015.11.024 26592951 PMC4876052

[B7] Beste C. Steenbergen L. Sellaro R. Grigoriadou S. Zhang R. Chmielewski W. (2016). Effects of concomitant stimulation of the gabaergic and norepinephrine system on inhibitory control – a study using transcutaneous vagus nerve stimulation. *Brain Stimul.* 9 811–818. 10.1016/j.brs.2016.07.004 27522167

[B8] Borges U. Laborde S. Raab M. (2019). Influence of transcutaneous vagus nerve stimulation on cardiac vagal activity: not different from sham stimulation and no effect of stimulation intensity. *PLoS One* 14:e0223848. 10.1371/journal.pone.0223848 31603939 PMC6788680

[B9] Burger A. M. van der Does W. Brosschot J. F. Verkuil B. (2020). From ear to eye? No effect of transcutaneous vagus nerve stimulation on human pupil dilation: a report of three studies. *Biol. Psychol.* 152:107863. 10.1016/j.biopsycho.2020.107863 32050095

[B10] Burger A. M. Van der Does W. Thayer J. F. Brosschot J. F. Verkuil B. (2019). Transcutaneous vagus nerve stimulation reduces spontaneous but not induced negative thought intrusions in high worriers. *Biol. Psychol.* 142 80–89. 10.1016/j.biopsycho.2019.01.014 30710565

[B11] Burger A. M. Verkuil B. Van Diest I. Van der Does W. Thayer J. F. Brosschot J. F. (2016). The effects of transcutaneous vagus nerve stimulation on conditioned fear extinction in humans. *Neurobiol. Learn. Mem.* 132 49–56. 10.1016/j.nlm.2016.05.007 27222436

[B12] Butt M. F. Albusoda A. Farmer A. D. Aziz Q. (2019). The anatomical basis for transcutaneous auricular vagus nerve stimulation. *J. Anat.* 236 588–611. 10.1111/joa.13122 31742681 PMC7083568

[B13] Colzato L. S. Jongkees B. J. de Wit M. van der Molen M. J. W. Steenbergen L. (2018a). Variable heart rate and a flexible mind: higher resting-state heart rate variability predicts better task-switching. *Cogn. Affect. Behav. Neurosci.* 18 730–738. 10.3758/s13415-018-0600-x 29713957 PMC6096636

[B14] Colzato L. S. Ritter S. M. Steenbergen L. (2018b). Transcutaneous vagus nerve stimulation (tVNS) enhances divergent thinking. *Neuropsychologia* 111 72–76. 10.1016/j.neuropsychologia.2018.01.003 29326067

[B15] Dajani D. R. Uddin L. Q. (2015). Demystifying cognitive flexibility: implications for clinical and developmental neuroscience. *Trends Neurosci.* 38 571–578. 10.1016/j.tins.2015.07.003 26343956 PMC5414037

[B16] De Couck M. Cserjesi R. Caers R. Zijlstra W. P. Widjaja D. Wolf N. (2017). Effects of short and prolonged transcutaneous vagus nerve stimulation on heart rate variability in healthy subjects. *Auton. Neurosci. Basic Clin.* 203 88–96. 10.1016/j.autneu.2016.11.003 28017263

[B17] Diamond A. (2013). Executive functions. *Annu. Rev. Psychol.* 64 135–168. 10.1146/annurev-psych-113011-143750.Executive 23020641 PMC4084861

[B18] Dietrich S. Smith J. Scherzinger C. Hofmann-Preiß K. Freitag T. Eisenkolb A. (2008). A novel transcutaneous vagus nerve stimulation leads to brainstem and cerebral activations measured by functional MRI. *Biomed. Tech.(Berl.)* 53 104–111. 10.1515/BMT.2008.022 18601618

[B19] Dwan K. Li T. Altman D. G. Elbourne D. (2019). CONSORT 2010 statement: extension to randomised crossover trials. *BMJ* 366:l4378 10.1136/bmj.l4378 PMC6667942 31366597

[B20] Ellrich J. (2011). Transcutaneous vagus nerve stimulation. *Eur. Neurol. Rev.* 6:254 10.17925/ENR.2011.06.04.254

[B21] Eriksen B. A. Eriksen C. W. (1974). Effects of noise letters upon the identification of a target letter in a nonsearch task. *Percept. Psychophys.* 16 143–149. 10.3758/BF03203267

[B22] Faul F. Erdfelder E. Lang A.-G. Buchner A. (2007). G^∗^Power 3: a flexible statistical power analysis program for the social, behavioral, and biomedical sciences. *Behav. Res. Methods* 39 175–191. 10.3758/BF03193146 17695343

[B23] Finisguerra A. Crescentini C. Urgesi C. (2019). Transcutaneous vagus nerve stimulation affects implicit spiritual self-representations. *Neuroscience* 412 144–159. 10.1016/j.neuroscience.2019.05.059 31176701

[B24] Fischer R. Ventura-Bort C. Hamm A. Weymar M. (2018). Transcutaneous vagus nerve stimulation (tVNS) enhances conflict-triggered adjustment of cognitive control. *Cogn. Affect. Behav. Neurosci.* 18 680–693. 10.3758/s13415-018-0596-2 29693214

[B25] Foote S. L. Bloom F. E. Aston-Jones G. (1983). Nucleus locus ceruleus: new evidence of anatomical and physiological specificity. *Physiol. Rev.* 63 844–914. 10.1152/physrev.1983.63.3.844 6308694

[B26] Frangos E. Ellrich J. Komisaruk B. R. (2015). Non-invasive access to the vagus nerve central projections via electrical stimulation of the external ear: FMRI evidence in humans. *Brain Stimul.* 8 624–636. 10.1016/j.brs.2014.11.018 25573069 PMC4458242

[B27] Jennings J. R. Allen B. Gianaros P. J. Thayer J. F. Stephen B. (2015). Focusing neurovisceral integration: cognition, heart rate variability, and cerebral blood flow. *Psychophysiology* 52 214–224. 10.1111/psyp.12319.Focusing 25160649 PMC4387874

[B28] Johnsen B. H. Thayer J. F. Laberg J. C. Wormnes B. Raadal M. Skaret E. (2003). Attentional and physiological characteristics of patients with dental anxiety. *J. Anxiety Disord.* 17 75–87. 12464290 10.1016/s0887-6185(02)00178-0

[B29] Keute M. Boehrer L. Ruhnau P. Heinze H.-J. Zaehle T. (2019a). Transcutaneous vagus nerve stimulation (tVNS) and the dynamics of visual bistable perception. *Front. Neurosci.* 13:227. 10.3389/fnins.2019.00227 30906250 PMC6418039

[B30] Keute M. Demirezen M. Graf A. Mueller N. G. Zaehle T. (2019b). No modulation of pupil size and event-related pupil response by transcutaneous auricular vagus nerve stimulation (taVNS). *Sci. Rep.* 9:11452. 10.1038/s41598-019-47961-4 31391505 PMC6685960

[B31] Keute M. Ruhnau P. Heinze H. Zaehle T. (2018a). Behavioral and electrophysiological evidence for GABAergic modulation through transcutaneous vagus nerve stimulation. *Clin. Neurophysiol.* 129 1789–1795. 10.1016/j.clinph.2018.05.026 29981954

[B32] Keute M. Ruhnau P. Zaehle T. (2018b). Reply to “Reconsidering sham in transcutaneous vagus nerve stimulation studies.”. *Clin. Neurophysiol.* 129 2503–2504. 10.1016/j.clinph.2018.09.001 30249501

[B33] Koenig J. Thayer J. F. (2016). Sex differences in healthy human heart rate variability?: a meta-analysis. *Neurosci. Biobehav. Rev.* 64 288–310. 10.1016/j.neubiorev.2016.03.007 26964804

[B34] Kraus T. Hösl K. Kiess O. Schanze A. Kornhuber J. Forster C. (2007). BOLD fMRI deactivation of limbic and temporal brain structures and mood enhancing effect by transcutaneous vagus nerve stimulation. *J. Neural Trans.* 114 1485–1493. 10.1007/s00702-007-0755-z 17564758

[B35] Kraus T. Kiess O. Hösl K. Terekhin P. Kornhuber J. Forster C. (2013). CNS BOLD fMRI effects of sham-controlled transcutaneous electrical nerve stimulation in the left outer auditory canal – a pilot study. *Brain Stimul.* 6 798–804. 10.1016/j.brs.2013.01.011 23453934

[B36] Laborde S. Mosley E. Thayer J. F. (2017). Heart rate variability and cardiac vagal tone in psychophysiological research – recommendations for experiment planning, data analysis, and data reporting. *Front. Psychol.* 08:213. 10.3389/fpsyg.2017.00213 28265249 PMC5316555

[B37] Liepelt R. Porcu E. Stenzel A. Lappe M. (2019). Saccadic eye movements do not trigger a joint Simon effect. *Psychon. Bull. Rev.* 26 1896–1904. 10.3758/s13423-019-01639-0 31347035

[B38] Malik M. Camm A. J. Bigger J. T. Breithart G. Cerutti S. Cohen R. (1996). Heart rate variability: standards of measurement, physiological interpretation and clinical use. Task force of the european society of cardiology and the North American society of pacing and electrophysiology. *Circulation* 93 1043–1065. 8598068

[B39] Marotta A. Román-Caballero R. Lupiáñez J. (2018). Arrows don’t look at you?: qualitatively different attentional mechanisms triggered by gaze and arrows. *Psychon. Bull. Rev.* 25 2254–2259. 10.3758/s13423-018-1457-2 29546665

[B40] Mayr U. Keele S. W. (2000). Changing internal constraints on action: the role of backward inhibition. *J. Exp. Psychol. Gener.* 129 4–26. 10.1037//0096-3445.129.1.4 10756484

[B41] Miyake A. Friedman N. P. (2012). The nature and organization of individual differences in executive functions: four general conclusions. *Curr. Dir. Psychol. Sci.* 21 8–14. 10.1177/0963721411429458 22773897 PMC3388901

[B42] Miyake A. Friedman N. P. Emerson M. J. Witzki A. H. Howerter A. Wager T. D. (2000). The unity and diversity of executive functions and their contributions to complex “‘ Frontal Lobe ”’ tasks?: a latent variable analysis. *Cogn. Psychol.* 41 49–100. 10.1006/cogp.1999.0734 10945922

[B43] Murray A. R. Atkinson L. Mahadi M. K. Deuchars S. A. Deuchars J. (2016). The strange case of the ear and the heart: the auricular vagus nerve and its influence on cardiac control. *Auton. Neurosci. Basic Clin.* 199 48–53. 10.1016/j.autneu.2016.06.004 27388046

[B44] Peirce J. Gray J. R. Simpson S. MacAskill M. Höchenberger R. Sogo H. (2019). PsychoPy2: experiments in behavior made easy. *Behav. Res. Methods* 51 195–203. 10.3758/s13428-018-01193-y 30734206 PMC6420413

[B45] Peuker E. T. Filler T. J. (2002). The nerve supply of the human auricle. *Clin. Anat.* 15 35–37. 10.1002/ca.1089 11835542

[B46] Quintana D. S. Alvares G. A. Heathers J. A. J. (2016). Guidelines for reporting articles on psychiatry and heart rate variability (GRAPH): recommendations to advance research communication. *Transl. Psychiatry* 6 e803–e810. 10.1038/tp.2016.73 27163204 PMC5070064

[B47] Rangon C.-M. (2018). Reconsidering sham in transcutaneous vagus nerve stimulation studies. *Clin. Neurophysiol.* 129 2501–2502. 10.1016/j.clinph.2018.08.027 30268709

[B48] Sara S. J. (2015). Locus coeruleus in time with the making of memories. *Curr. Opin. Neurobiol.* 35 87–94. 10.1016/j.conb.2015.07.004 26241632

[B49] Sellaro R. Gelder B. De Finisguerra A. Colzato L. S. (2017). Transcutaneous vagus nerve stimulation (tVNS) enhances recognition of emotions in faces but not bodies. *Cortex* 99 213–223. 10.1016/j.cortex.2017.11.007 29275193

[B50] Smith R. Thayer J. F. Khalsa S. S. Lane R. D. (2017). The hierarchical basis of neurovisceral integration. *Neurosci. Biobehav. Rev.* 75 274–296. 10.1016/j.neubiorev.2017.02.003 28188890

[B51] Steenbergen L. Sellaro R. Stock A.-K. Verkuil B. Beste C. Colzato L. S. (2015). Transcutaneous vagus nerve stimulation (tVNS) enhances response selection during action cascading processes. *Eur. Neuropsychopharmacol.* 25 773–778. 10.1016/j.euroneuro.2015.03.015 25869158

[B52] Tafuro A. Ambrosini E. Puccioni O. Vallesi A. (2019). Brain oscillations in cognitive control: a cross-sectional study with a spatial stroop task. *Neuropsychologia* 133:107190. 10.1016/j.neuropsychologia.2019.107190 31513806

[B53] Tarvainen M. P. Niskanen J. Lipponen J. A. Ranta-aho P. O. Karjalainen P. A. (2013). Kubios HRV – heart rate variability. *Comput. Methods Programs Biomed.* 113 210–220. 10.1016/j.cmpb.2013.07.024 24054542

[B54] Thayer J. F. Åhs F. Fredrikson M. Sollers J. J. Wager T. D. (2012). A meta-analysis of heart rate variability and neuroimaging studies: implications for heart rate variability as a marker of stress and health. *Neurosci. Biobehav. Rev.* 36 747–756. 10.1016/j.neubiorev.2011.11.009 22178086

[B55] Thayer J. F. Hansen A. L. Saus-Rose E. Johnsen B. H. (2009). Heart rate variability, prefrontal neural function, and cognitive performance: the neurovisceral integration perspective on self-regulation, adaptation, and health. *Ann. Behav. Med.* 37 141–153. 10.1007/s12160-009-9101-z 19424767

[B56] van Leusden J. W. R. Sellaro R. Colzato L. S. (2015). Transcutaneous vagal nerve stimulation (tVNS): a new neuromodulation tool in healthy humans? *Front. Psychol.* 6:102 10.3389/fpsyg.2015.00102 PMC4322601 25713547

[B57] Ventura-Bort C. Wirkner J. Genheimer H. Wendt J. Hamm A. O. Weymar M. (2018). Effects of transcutaneous vagus nerve stimulation (tVNS) on the P300 and alpha-amylase level: a pilot study. *Front. Hum. Neurosci.* 12:202. 10.3389/fnhum.2018.00202 29977196 PMC6021745

[B58] Wagenmakers E.-J. (2007). A practical solution to the pervasive problems of p values. *Psychon. Bull. Rev.* 14 779–804. 10.3758/BF03194105 18087943

[B59] Walker R. M. James O. Brewer G. A. (2017). Replication, experiments and knowledge in public management research. *Public Manag. Rev.* 19 1221–1234. 10.1080/14719037.2017.1282003 31753271

[B60] Warren C. M. Tona K. D. Ouwerkerk L. van Paridon J. Poletiek F. Bosch J. A. (2018). The neuromodulatory and hormonal effects of transcutaneous vagus nerve stimulation as evidenced by salivary alpha amylase, salivary cortisol, pupil diameter, and the P3 event-related potential. *Brain Stimul.* 12 635–642. 10.1016/j.brs.2018.12.224 30591360

[B61] Wetzels R. Matzke D. Lee M. D. Rouder J. N. Iverson G. J. Wagenmakers E. (2011). Statistical evidence in experimental psychology: an empirical comparison using 855 t Tests. *Perspect. Psychol. Sci. ?* 6 291–298. 10.1177/1745691611406923 26168519

[B62] Yakunina N. Kim S. S. (2017). Optimization of transcutaneous vagus nerve stimulation using functional MRI. *Neuromodulation* 20 290–300. 10.1111/ner.12541 27898202

[B63] Zelazo P. D. Anderson J. E. Richler J. Wallner-Allen K. Beaumont J. L. Conway K. P. (2014). NIH toolbox cognition battery (CB): validation of executive function measures in adults. *J. Int. Neuropsychol. Soc.* 20 620–629. 10.1017/S1355617714000472 24960301 PMC4601803

