# Supplementary material for: Transcutaneous Vagus Nerve Stimulation May Enhance Only Specific Aspects of the Core Executive Functions. A Randomized Crossover Trial
Source: Front Neurosci. 2020 May 25;14:523. doi: 10.3389/fnins.2020.00523 (PMC7262369; doi:10.3389/fnins.2020.00523)
Supplement: Supplementary file 1 [file Table_1.docx]

**Supplementary material – data sheet 1**

**Estimation of carry-over effects on RMSSD and HF.** Two separated repeated measures analyses of variance (rmANOVAs) for root mean square of successive differences (RMSSD) and for high frequency (HF), respectively, were performed to check if the resting phases, namely the first, the second, the third, and the fourth resting phases, differed from each other regarding RMSSD and HF when arranged chronologically. We also took the testing days (Day 1 and Day 2) into account to check if there was a difference in RMSSD and HF from the first to the second day. The different RMSSD measurements during the resting phases differed significantly from each other, *F*(3, 96) = 38.660, *p* < .001, η_p_² = .547 (Fig. S1). Six post hoc tests (Bonferroni-corrected *p* = .008) revealed that there was a significant increase of RMSSD between Resting Phase 1 (*M* = 43.35, *SD* = 16.74) and Resting Phase 2 (*M* = 48.87, *SD* = 19.31), *t*(32) = 3.852, *p*< .001, *d* = 0.671. Resting Phase 3 (*M* = 55.63, *SD* = 21.00) was significantly higher than Resting Phase 1, *t*(32) = 5.804, *p*< .001, *d* = 1.010, and Resting Phase 2, *t*(32) = 3.173, *p*= .003, *d* = 0.552. Resting Phase 4 (*M* = 61.14, *SD* = 21.85) was significantly higher than Resting Phase 1, *t*(32) = 8.660, *p*< .001, *d* = 1.508. Resting Phase 4 was significantly higher than Resting Phase 2, *t*(30) = 6.799, *p*< .001, *d* = 1.184. Further, the different testing days did not differ significantly from each other regarding RMSSD, *F*(1, 32) = 0.016, *p* = .899. Regarding HF, there was neither an effect of sequence of resting phases, *F*(3, 96) = 50.260, *p* = .093, nor of testing day, *F*(1, 32) = 0.126, *p* = .725.


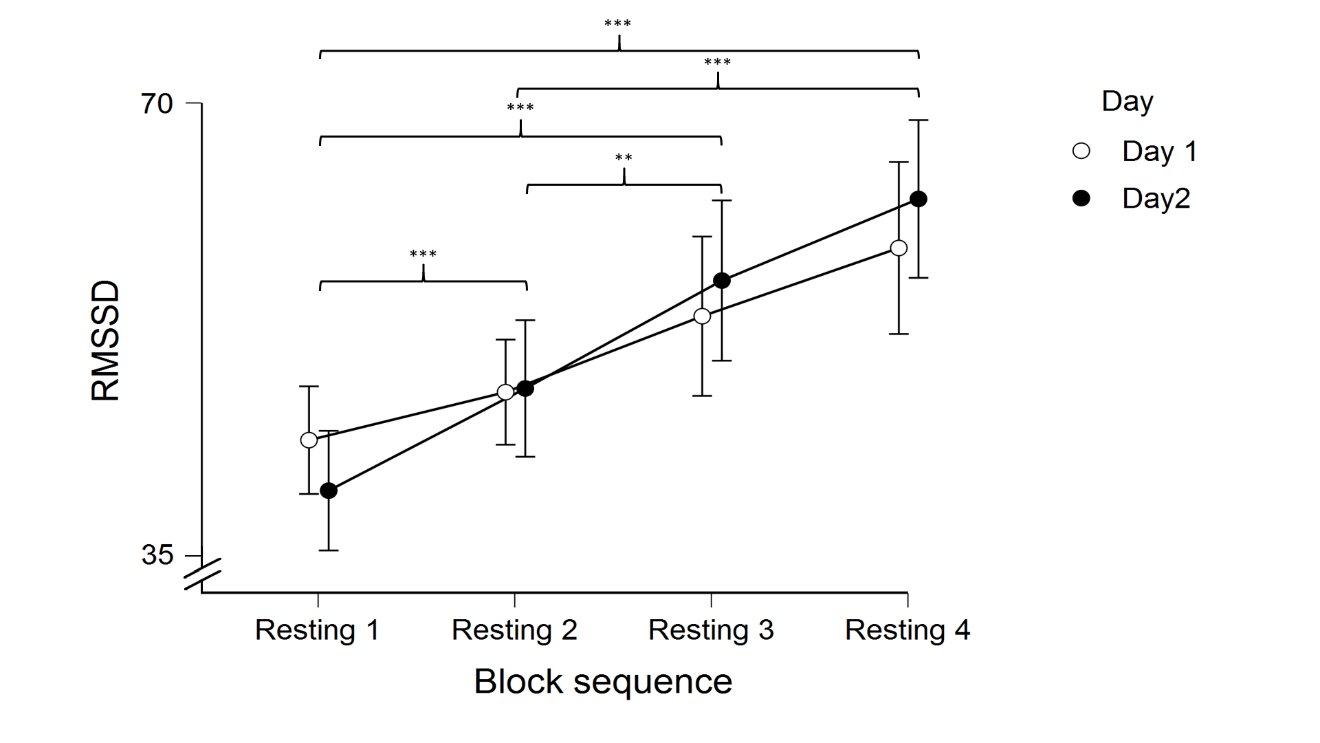


Figure S1. Mean scores of root mean square of successive differences (RMSSD). Scores during the resting phases from each block put in chronological order. Error bars represent confidence intervals (95%). **p* < .05, ***p* < .01, ****p* < .001.

**Estimation of learning effects on cognitive measurements.** We ran 2 (Day 1 and Day 2) x 2 (congruency or trial type, depending on the task) rmANOVAs as well as paired samples t-tests (only for switch costs) for all measurements considered for the four cognitive tasks used in the present study. These tests were performed to check if there was an improvement in task performance, i.e., faster reaction times (RT), lower percentage error, and lower switch costs, from Testing Day 1 to Testing Day 2. We calculated paired samples t-tests for collapsed means in case an effect could be found. Because post-hoc tests for the main effects consist of only one t-test, given the 2x2 design, Bonferroni corrections were performed only for potential interaction effects.

In the Flanker task, there was an effect of congruency on RT, *F*(1, 31) = 242.683, *p* < .001, η_p_² = .890. The post-hoc test revealed that RT in incongruent trials (*M* = 557.71 ms, *SD* = 63.104) was significantly higher than RT in congruent trials (*M* = 511.60 ms, *SD* = 56.28), *t*(31) = 13.062, *p* < .001, *d* = 2.346. There was no effect of day, *F*(1, 31) = 0.006, *p* = .937, and no interaction effect of day with congruency, *F*(1, 31) = 2.145, *p* = .153. Regarding percentage error in the Flanker task, there was an effect of congruency, *F*(1, 31) = 127.821, *p* < .001, η_p_² = .810, with the post-hoc test revealing that percentage error in incongruent trials (*M* = 8.50% *SD* = 8.04) was significantly higher than in congruent trials (*M* = 0.70%, *SD* = 0.93), *t*(31) = 5.841, *p* < .001, *d* = 1.049. There was no effect of day, *F*(1, 31) = 0.704, *p* = .408, and no interaction effect, *F*(1, 31) = 0.094, *p* = .761.

In the Spatial Stroop task, there was an effect of congruency on RT, *F*(1, 31) = 55.599, *p* < .001, η_p_² = .650. The post-hoc test revealed that RT in incongruent trials (*M* = 531.212 ms, *SD* = 52.499) was significantly higher than in congruent trials (*M* = 515.83 ms, *SD* = 50.70), *t*(31) = 5.580, *p* < .001, *d* = 1.002. There was no effect of day, *F*(1, 31) = 0.027, *p* = .872, and no interaction effect, *F*(1, 31) = 0.426, *p* = .519. Regarding percentage error, there was an effect of congruency, *F*(1, 31) = 34.242, *p* < .001, η_p_² = .533. The post-hoc test revealed that percentage error in incongruent trials (*M* = 4.10%, *SD* = 3.62) was significantly higher than in congruent trials (*M* = 1.31%, *SD* = 1.41), *t*(31) = 4.972, *p* < .001, *d* = 0.892. There was no effect of day, *F*(1, 31) = 0.293, *p* = .592, and no interaction effect, *F*(1, 31) = 0.405, *p* = .529.

In the NLT, there was an effect of trial type on RT, *F*(1, 31) = 220.646, *p* < .001, η_p_² = .877, with RT in the non-switch trials (*M* = 961.61 ms, *SD* = 126.67) being significantly faster than the RT in the switch trials (*M* = 1,202.21 ms, *SD* = 127.88), *t*(31) = 14.803, *p* < .001, *d* = 2.617. There was no effect of day, *F*(1, 31) = 0.265, *p* = .610, and no interaction effect, *F*(1, 31) = 1.856, *p* = .183. Regarding percentage error in the NLT, there was an effect of trial type, *F*(1, 31) = 71.954, *p* < .001, η_p_² = .699, with the post-hoc test revealing that percentage error in switch trials (*M* = 25.53%, *SD* = 13.51) was significantly higher than in non-switch trials (*M* = 18.14%, *SD* = 12.75), *t*(31) = 9.571, *p* < .001, *d* = 1.692. There was no effect of day, *F*(1, 31) = 1.082, *p* = .306, and no interaction effect, *F*(1, 31) = 0.558, *p* = .461. Regarding switch costs, there was no significant difference between Day 1 and Day 2, *t*(31) = 1.141, *p* = .263.

In the DCCS, there was no effect of trial type on RT, *F*(1, 31) = 1.068, *p* = .310, no effect of day, *F*(1, 31) = 0.004, *p* = .951, and no interaction effect, *F*(1, 31) = 1.105, *p* = .301. Regarding percentage error, there was an effect of trial type, *F*(1, 31) = 11.943, *p* < .001, η_p_² = .285, with the post-hoc test revealing that percentage error in switch trials (*M* = 2.78%, *SD* = 1.83) was significantly higher than in non-switch trials (*M* = 2.19%, *SD* = 1.83), *t*(31) = 3.862, *p* < .001, *d* = 0.694. There was no effect of day, *F*(1, 31) = 2.162, *p* = .152, and no interaction effect, *F*(1, 31) = 0.683, *p* = .415. In the DCCS, switch costs on Day 1 (*M* = 20.66 ms, *SD* = 39.74) were significantly higher than on Day 2 (*M* = -7.14 ms, *SD* = 51.04), *t*(31) = 2.151, *p* = .040, *d* = 0.386.

To sum up, in line with the main results of the present study, conflict effects could be observed in the Flanker task, the Spatial Stroop task, in the DCCS, and in the NLT. Importantly, in all tasks neither RT nor the percentage error changed significantly from Day 1 to Day 2 of testing. Similar with the main analysis, switch costs in the NLT did not change significantly, whereas switch costs in the DCCS increased from Day 1 to Day 2, thus pointing to a learning effect specifically in this parameter.

**Estimation of novelty effects on cognitive measurements.** To check whether tVNS affects task performance more strongly when its trials are novel, we split the trials of the tasks into first and second half, whereby first half would correspond to novel trials, and collapsed the congruent/non-switch with the incongruent/switch trials. We then ran 2x2 rmANOVAs with stimulation (active and sham stimulation) and novelty (first and second half of the task) as factors, and RT and percentage error of all tasks as dependent variables. We calculated paired samples t-tests for collapsed means in case an effect could be found. Because post-hoc tests for the main effects consist of only one t-test, given the 2x2 design, Bonferroni corrections were performed only for potential interaction effects.

In the Flanker task, there was an effect of novelty regarding RT, *F*(1, 31) = 8.826, *p* = .006, η_p_² = .222. The post-hoc test revealed that RT in the first half of the task (*M* = 508.46 ms, *SD* = 55.83) was significantly lower than RT in the second half of the task (*M* = 521.09 ms, *SD* = 62.75), *t*(31) = 2.971, *p* = .006, *d* = 0.525. There was no effect of stimulation, *F*(1, 31) = 0.724, *p* = .401, and no interaction effect of novelty with stimulation, *F*(1, 31) = 3.217, *p* = .083. Regarding percentage error in the Flanker task, there was an effect of novelty, *F*(1, 31) = 239.719, *p* < .001, η_p_² = .885, with the post-hoc test revealing that percentage error in the first half of the task (*M* = 4.46% *SD* = 4.44) was significantly lower than in the second half of the task (*M* = 9.58%, *SD* = 6.59), *t*(31) = 15.483, *p* < .001, *d* = 2.737. There was no effect of stimulation, *F*(1, 31) = 0.782, *p* = .383, and no interaction effect, *F*(1, 31) = 2.152, *p* = .152.

In the Spatial Stroop task, there was no effect of novelty on RT, *F*(1, 31) = 2.779, *p* = .106. There was no effect of stimulation, *F*(1, 31) = 0.653, *p* = .425, and no interaction effect, *F*(1, 31) = 0.009, *p* = .924. Similarly, regarding percentage error there was no effect of congruency, *F*(1, 31) = 0.510, *p* = .481, of stimulation, *F*(1, 31) = 0.084, *p* = .773, and no interaction effect, *F*(1, 31) = 2.125, *p* = .155.

In the NLT, there was no effect of novelty on RT, *F*(1, 31) = 1.588, *p* = .217. There was no effect of stimulation, *F*(1, 31) = 0.453, *p* = .506, and no interaction effect, *F*(1, 31) = 2.092, *p* = .158. Regarding percentage error in the NLT, there was an effect of novelty, *F*(1, 31) = 116.663, *p* < .001, η_p_² = .790, with the post-hoc test revealing that percentage error in the first half of the task (*M* = 22.88%, *SD* = 3.19) was significantly higher than in the second half of the task (*M* = 20.33%, *SD* = 2.79), *t*(31) = 10.801, *p* < .001, *d* = 1.909. There was no effect of stimulation, *F*(1, 31) = 1.948, *p* = .173, and no interaction effect, *F*(1, 31) = 0.004, *p* = .948.

In the DCCS, there was an effect of novelty on RT, *F*(1, 31) = 8.413, *p* = .007, η_p_² = 0.213, with the post-hoc test revealing that RT in the first half of the task (*M* = 583.51 ms, *SD* = 88.42) was significantly lower than in the second half (*M* = 613.01, *SD* = 114.70), *t*(31) = 2.900, *p* = .007, *d* = 0.513. No effect of stimulation on RT, *F*(1, 31) = 0.033, *p* = .856, and no interaction effect, *F*(1, 31) = 0.904, *p* = .349, could be found. Regarding percentage error, there was no effect of novelty, *F*(1, 31) = 1.222, *p* = .278, of stimulation, *F*(1, 31) = 0.116, *p* = .736, and no interaction effect, *F*(1, 31) = 2.964, *p* = .095.

To sum up, cognitive performance changed from the first to second task half in the most of the cognitive tasks, but tVNS did not affect novel trials more strongly than the less novel ones. Furthermore, the change in task performance was different between the tasks. In the inhibition tasks, there was a decreased RT and percentage error in the Flanker task, but no changes in the Spatial Stroop task. In the cognitive flexibility tasks, less errors could be detected across the NLT, and higher RT in the second half of the DCCS was found. Thus, a novelty effect could not be found in the present study.
